# Structure and function of the Toscana virus cap-snatching endonuclease

**DOI:** 10.1093/nar/gkz838

**Published:** 2019-10-04

**Authors:** Rhian Jones, Sana Lessoued, Kristina Meier, Stéphanie Devignot, Sergio Barata-García, Maria Mate, Gabriel Bragagnolo, Friedemann Weber, Maria Rosenthal, Juan Reguera

**Affiliations:** 1 Aix-Marseille Université, CNRS, AFMB UMR 7257, 13288 Marseille, France; 2 INSERM, AFMB UMR7257,13288 Marseille, France; 3 Bernhard Nocht Institute for Tropical Medicine, Department of Virology, D-20359 Hamburg, Germany; 4 Institute for Virology, FB10-Veterinary Medicine, Justus-Liebig University, D-35392 Giessen, Germany

## Abstract

Toscana virus (TOSV) is an arthropod-borne human pathogen responsible for seasonal outbreaks of fever and meningoencephalitis in the Mediterranean basin. TOSV is a segmented negative-strand RNA virus (sNSV) that belongs to the genus phlebovirus (family *Phenuiviridae*, order Bunyavirales), encompassing other important human pathogens such as Rift Valley fever virus (RVFV). Here, we carried out a structural and functional characterization of the TOSV cap-snatching endonuclease, an N terminal domain of the viral polymerase (L protein) that provides capped 3′OH primers for transcription. We report TOSV endonuclease crystal structures in the apo form, in complex with a di-ketoacid inhibitor (DPBA) and in an intermediate state of inhibitor release, showing details on substrate binding and active site dynamics. The structure reveals substantial folding rearrangements absent in previously reported cap-snatching endonucleases. These include the relocation of the N terminus and the appearance of new structural motifs important for transcription and replication. The enzyme shows high activity rates comparable to other His+ cap-snatching endonucleases. Moreover, the activity is dependent on conserved residues involved in metal ion and substrate binding. Altogether, these results bring new light on the structure and function of cap-snatching endonucleases and pave the way for the development of specific and broad-spectrum antivirals.

## INTRODUCTION

Toscana virus (TOSV) is a member of the genus phlebovirus (family *Phenuiviridae*, order *Bunyavirales*), that includes several arthropod-borne human pathogens such as Sandfly fever sicilian virus (SFSV), Severe fever with thrombocytopenia syndrome virus (SFTSV) or Rift Valley fever virus (RVFV), that cause outbreaks in the Mediterranean basin, Asia, and sub-Saharan Africa, respectively. Symptoms range from mild to severe central nervous system infections (TOSV, SFSV) up to hemorrhagic fevers (SFTSV, RVFV) (1–3). The absence of efficient drugs and vaccines for these emerging pathogens means that there is an urgent need for research and development of tools for their diagnostics and treatment.

Like all segmented negative-strand RNA viruses (sNSVs), *Phenuiviridae* family members use a ‘cap-snatching’ mechanism to initiate transcription of the viral genome. By this mechanism, host cell messenger RNAs (mRNAs) are sequestered and cleaved by the EN around 11 nucleotides downstream of the capped 5′ end. The resulting small capped RNA fragments are then used for the initiation of viral transcription by the L protein RNA-dependent RNA polymerase (RdRp) domain. Cap-snatching has been extensively structurally and biochemically characterized for influenza virus, which replicates in the cell nucleus and has a heterotrimeric polymerase instead of the monomeric L protein present in bunyaviruses (4). The comparison of the structures of influenza A and B polymerases illustrated the possible mechanics underlying this process; whereby the cap binding domain (in the PB2 subunit), captures the cellular mRNA and orientates it towards the EN domain (at the N terminus of the PA subunit) for its cleavage before re-directing the RNA into the RdRp catalytic site (in the PB1 subunit) for priming transcription initiation (5–7). Bunyavirales carry out cap-snatching by their multifunctional L protein, which harbours the EN at the N terminus. There are no L protein EN structures available for any *Phenuiviridae* family members, and certain lines of evidence suggest that the cap-snatching processes may differ among the sNSVs. Firstly, considering the similar architecture of sNSV polymerases reported to date (8), for arenaviruses the length of the host-derived 5′ mRNA fragments (5–6 nucleotides (9–11)) is too short to allow transcription priming via the mechanism proposed for influenza virus (requiring fragments of 10 to 15 nucleotides (12)). This is even more remarkable for the influenza-related Thogotoviruses (THOV; family *Orthomyxoviridae*), that contains only one heterologous nucleotide in the 5′ end of their mRNAs (13,14). Additionally, the sources of mRNA in the nucleus (where influenza virus replicates and transcribes) are different from those in the cytoplasm (where bunyaviruses replicate and transcribe). In conclusion, structural and/or mechanistic variations in the influenza virus cap-snatching mechanism occur among sNSVs and still need to be understood.

The presence of a cap-snatching EN is a common feature among the sNSV polymerases. The first structural and functional characterization of a cap-snatching EN was reported for influenza virus (15,16). These structures revealed that the EN belongs to the metal ion dependent PD-D/ExK nuclease superfamily, including many different enzymes involved in various aspects of DNA metabolism (e.g. EcoRV). The motif includes acidic residues responsible for metal ion coordination, and a lysine responsible for the stabilisation and orientation of the attacking water hydroxide nucleophile for hydrolysis of the RNA phosphate backbone (17). Shortly after, cap-snatching EN structures for the La Crosse virus (LACV, family *Peribunyaviridae*) and the Lymphocytic choriomeningitis virus (LCMV, family *Arenaviridae*) were isolated and structurally characterised, demonstrating the general presence of a (PD-D/ExK) superfamily of cation-dependent endonucleases in the N terminus of the sNSV L proteins (18,19). Exceptions are the super large L proteins of tenuiviruses or nairoviruses, that include additional domains in the N terminus (20). Mutation of the EN active site residues in full-length L proteins proved to abrogate the cap-snatching based transcription in reconstituted minireplicon studies (18,21–23).

A comparative study including influenza virus, arenavirus and bunyaviruses (the last two recently included in the new order *Bunyavirales* (24)), reported that some cap-snatching ENs, when isolated from the full-length polymerase, are active *in vitro* while some others are not. The presence of a catalytic histidine in the active site determines their high activity rates by favouring the canonical binding of the catalytic metal ions in the active site. Based on this evidence, a classification of cap-snatching ENs was proposed as His+ (active *in vitro*) and His– (inactive *in vitro*), permitting prediction of the activity of a cap-snatching EN for functional studies *in vitro* and for screening of inhibitory compounds (25). These findings suggest that His- ENs are only active in the context of the full-length L protein in order to perform cap-snatching, indicating that different mechanisms of regulation (activation and inhibition) of EN activity may exist among sNSVs. Furthermore, the structure and the functional characterization of THOV cap-snatching EN revealed that, despite strong structural homology with influenza virus, the enzyme lacks essential residues of the catalytic motif and consequently lacks EN activity. This suggests that THOV has possibly evolved an alternative, and still elusive, strategy to gain access to the cap structure required for their mRNAs (26).

Besides the conserved residues defining their active sites, the cap-snatching ENs of the different sNSV families have no apparent sequence similarity (25). This prevents transfer of the available structural information between families. Consequently, there is a gap in our knowledge regarding structure-to-function relationships of cap-snatching ENs for many important human pathogens. This is the case for all members of the *Nairoviridae* and *Phenuiviridae* families of the order *Bunyavirales*. Here, we present the crystal structure of the *Phenuiviridae* member TOSV cap-snatching EN in the apo form and in complex with the di-ketoacid inhibitor 2,4-dioxo-4-phenylbutanoic acid (DPBA). We characterize its enzymatic activity, specificity and the importance of each catalytic residue in the reaction. We identify new residues involved in substrate binding and investigate their role in catalysis. The structure reveals new features exclusive to *Phenuiviridae* family that we show have functional implications beyond transcription. We discuss the implications of these new structural features for the cap-snatching mechanism in the context of the full-length L proteins and for the evolution of the super large L proteins found in tenuiviruses and nairoviruses.

## MATERIALS AND METHODS

### Cloning and mutagenesis

The cDNA of the TOSV virus L protein (strain France AR_2005) coding for the first N terminal 233 amino acids (as reported in (19) and kindly provided by Bruno Coutard through the EVAg consortium), was cloned in pETG20A as an N terminal Thioredoxin-Hexahistidine fusion followed by a Tobacco Etch Virus protease (TEVpro) cleavage site. Shorter constructs with lengths of 221, 211, 201 and 191 aa were produced by In-fusion cloning following the supplier's protocols (Clontech) in Top 10 super competent *Escherichia coli* cells. Site directed mutagenesis of the 211 residues long construct (NTos211) was performed by the quick-change method using CloneAmp Hifi polymerase (Clontech). All constructs and mutants where validated by Sanger sequencing.

### Protein expression and purification

Proteins were expressed in *E. coli* strain BL21 (DE3) in TB media with 50 μM kanamycin at 17°C overnight after induction with 0.5 mM of IPTG. Selenomethionine labelled protein was obtained by expressing the NTos211 protein construct in *E. coli* BL21 cells in M9 minimal medium containing 50 mg/l of seleno-methionine (SeMet). The cells were disrupted by sonication on ice for 3 min in lysis buffer (50 mM Tris–HCl pH8, 300 mM NaCl, glycerol 5%, 10 mM imidazole and 2 mM TCEP) with EDTA-free protease inhibitor cocktail (Roche). The protein from the soluble fraction was loaded onto a 5–10 ml chelating sepharose column with nickel, washed with 5 column volumes of lysis buffer containing 10 mM imidazole, 2 column volumes with 40 mM imidazole and eluted with 5 volumes of lysis buffer with 400 mM imidazole. The eluted protein was cleaved with histidine tagged TEV protease overnight at 4°C during dialysis against lysis buffer, yielding an additional glycine before the second amino acid residue of the original sequence. A second nickel column step was performed to remove unwanted material. The resulting untagged proteins were concentrated and purified by size-exclusion chromatography using Superdex 75 or 200 columns (GE Healthcare) with 10 mM HEPES pH 7.6, 150 mM NaCl and 2 mM TCEP.

### Thermal stability experiments and EN activity assays

The influence of divalent metal ions and DPBA binding on protein stability was measured by thermal stability assays (TSA) (27), using a BioRad real-time PCR machine. The protein was incubated at 25 μM in assay buffer (35 mM Tris–HCl pH7.5, 150 mM NaCl, 2 mM TCEP) with different divalent metal cations at a concentration of 2 mM (with chloride as a counter ion), or with DPBA at a concentration of 0.5 mM. Curves were analysed in Prism, where melting temperature (*T*_m_) values were extracted from Boltzmann sigmoidal fits and are calculated from a minimum of three independent experiments, where readings were performed in triplicate.

For nuclease activity experiments, 2–10 μM of the EN was incubated with 0.5 μM of GA (FAM-5′-GAAAGAGCA_4_GGGA_9_) or CU RNA (FAM-5′-CU_4_CUCU_4_GCCCU_7_) labelled with a 5′ FAM (Microsynth) at room temperature for 1 hour in assay buffer, in the presence or absence of 2 mM Mn^2+^ or 0.5 mM DPBA. As cleavage of the GA RNA appeared to be more efficient, this RNA was selected for all experiments performed with mutants and N terminally truncated proteins. The reaction was stopped by adding a Tris–borate–EDTA loading buffer containing 8 M urea, and heating for one minute at 95°C. Reaction products were loaded onto 20% acrylamide 8 M urea TBE gels and the fluorescence visualized in a Typhoon imager (GE Healthcare). Experiments were performed independently at least three times for confirming reproducibility.

For the FRET based real-time quantitative EN activity assays, 500 nM of synthetic double labeled RNA with a 5′ fluorescent probe (6-FAM) and a 3′ quencher (BHQ1), 6-FAM-5′-CUCCUCAUUUUUCCCUAGUU-3′-BHQ1 (IBA), was mixed with the EN proteins as outlined previously (25) in a reaction buffer containing 20 mM Tris–HCl pH 8, 150 mM NaCl, 1 mM TCEP and 2 mM MnCl_2_. The fluorescence increase upon the RNA cleavage was measured in a TECAN (infinite M200 pro) at 26°C using wavelengths of 465 nm for excitation and 520 nm for emission. The initial reactions velocities were determined from the slope of the linear part of the reaction, where the fitting quality for a straight line was above *R*^2^ = 0.99.

### Crystallization

Crystallization trials with the TOSV EN native and SeMet derivatised proteins were performed by vapour diffusion in Swissci plates using a mosquito robot (TTP Labtech). Proteins in 10 mM HEPES pH 7.5, 150 mM NaCl, 2 mM TCEP and 2 mM MnCl_2_ were concentrated to 20 mg/ml and combined with the reservoir solution on a 1:1 ratio in 100 nl drops. The NTos211 construct crystallized in 100 mM HEPES pH 7.5 and 0.75 M LiSO_4_ as small bars. Co-crystallization experiments with the DPBA inhibitor at 0.2 mM concentration yielded robust thick plates in the same condition but at a lower protein concentration (5 mg/ml). The crystals grew at 20°C for between 48 h to one week and were frozen with mother liquor supplemented with 30% glycerol and 2 mM MnCl_2_. For the DPBA co-crystals, 0.5 mM DPBA was also added to the cryoprotectant.

### Crystallography

The TOSV EN structure was solved from data collected for the NTos211 SeMet DPBA co-crystals using single anomalous diffraction (SAD). Data were collected on beamline PROXIMA-1 (Soleil) at a wavelength of 0.979 Å (the selenium absorption maximum). Data were processed with XDS and solved with CRANK (28) within the CCP4i package. The TOSV EN protein crystallised in space group *p*2_1_2_1_2, with two molecules/monomers per asymmetric unit (asu). CRANK identified six anomalous sites within the asu, four with high estimated occupancies corresponding to the two SeMet residues per TOSV EN monomer, and another two sites with low estimated occupancies corresponding to two manganese ions in the active site of one of the two monomers. These sites permitted phasing the structure and, after density modification, generation of an experimental map for automatic model building by Buccaneer (29) that was extended by manual model building in Coot (30). Anomalous signal for two extra Mn^2+^ metal ions appeared in the anomalous density maps calculated with the final model built. For the final structure refinement, the data were reprocessed as native using the STARANISO server (http://staraniso.globalphasing.org/cgi-bin/staraniso.cgi) for the correction of anisotropy, which improved the refinement statistics and electron density maps. The structure was refined up to 2 Å resolution.

The data for the apo and DPBA-off crystals were collected at the ESRF in beamline id30a, reaching 2.4 and 1.5 Å resolution, respectively. Both crystallised in space group *p*2_1_2_1_2 with similar cell dimensions to the derivatised DPBA bound crystals (see Supplementary Table S1). The structures were solved by molecular replacement using PHASER. All structure refinement was performed iteratively with Phenix (31) and manual model building in Coot (30). Residues and sidechains without defined densities in the 2*F*_c_*F*_o_ calculated maps where removed from the final models. If not stated differently, PyMOL was used for visualization of all structures, generation of figures and the calculation of the root mean square deviations (RMSD) between the crystal structures by Rms-Cur.

### Cell-based RVFV minireplicon system experiments

L protein mutants were tested for their activity via the T7 RNA polymerase-based RVFV ambisense minireplicon system as described by Jérôme *et al.* (32). Mutagenic PCR with pCITE-RVFV-L as a template was used to generate L gene mutants. The presence of the desired mutation was confirmed by Sanger sequencing. The PCR products containing the functional cassette for expression of mutant L proteins were directly used for transfection after purification and spectrophotometric quantification without prior cloning. BSR-T7/5 cells stably expressing T7 RNA polymerase (33) were transfected with 250 ng of L gene PCR product, 750 ng of RVFV minigenome plasmid (pCITE-MG) expressing Renilla luciferase (Ren-Luc), 500 ng of pCITE-N expressing the nucleoprotein N, and 10 ng of pCITE-FF expressing firefly luciferase as a transfection control, per well in a 24-well plate using Lipofectamine 2000 (Thermo Fisher). One day post-transfection the cells were lysed in 100 μl passive lysis buffer (Promega) per well, and firefly luciferase as well as Ren-Luc activity were assessed using the dual-luciferase reporter assay system (Promega). Standardized relative light units (sRLU) were obtained by correcting Ren-Luc measurements with firefly luciferase levels to compensate for possible discrepancies in transfection efficiency and cell density.

For northern blot analysis, cells were transfected as stated above and total RNA was purified using the RNeasy Mini Kit (Qiagen) one day post-transfection. 750 ng of RNA was separated in a 1.5%-agarose-formaldehyde gel and transferred onto a Roti-Nylon plus membrane (Roth). Blots were hybridized with an antisense riboprobe labelled with ^32^P and targeting the Ren-Luc gene. Bands were visualized by autoradiography using a Typhoon scanner (GE Healthcare). For quantification of bands we used ImageJ2 software.

To confirm that all L protein mutants could be expressed in cells we performed transfection of BSR-T7/5 cells with 500 ng of PCR product expressing C terminally 3xFLAG-tagged versions of the respective L protein mutants per well in a 24-well plate using Lipofectamine 2000 (Thermo Fisher). Expression was enhanced by inoculating the cells prior to transfection with Modified Vaccinia virus Ankara expressing T7 RNA polymerase (34) at a multiplicity of infection of ∼3 for 1 h. At 24 h post transfection cytoplasmic lysates were separated in a 3–8% Tris-acetate polyacrylamide gel and transferred to a nitrocellulose membrane. The membrane was incubated with a peroxidase-conjugated anti-FLAG M2 antibody (1:10 000) (A8592; Sigma-Aldrich) for 1 h at room temperature and L protein bands were detected using the SuperSignal West Pico substrate (Pierce) and a FUSION SL image acquisition system (Vilber Lourmat).

## RESULTS

### Divalent cation dependent activity and stabilization of the Toscana cap-snatching endonuclease

In order to define the Toscana EN domain boundaries, we expressed a collection of C terminal deletion constructs including the first 233, 221, 211, 201, 191 and 181 amino acids of the TOSV L protein preceded by a histidine tagged thioredoxin domain linked by a TEVpro cleavage site (see methods). The shortest construct yielding soluble protein was 211 amino acid residues long (NTos211). We used this construct for subsequent biochemical and structural analysis.

**Figure 1.**
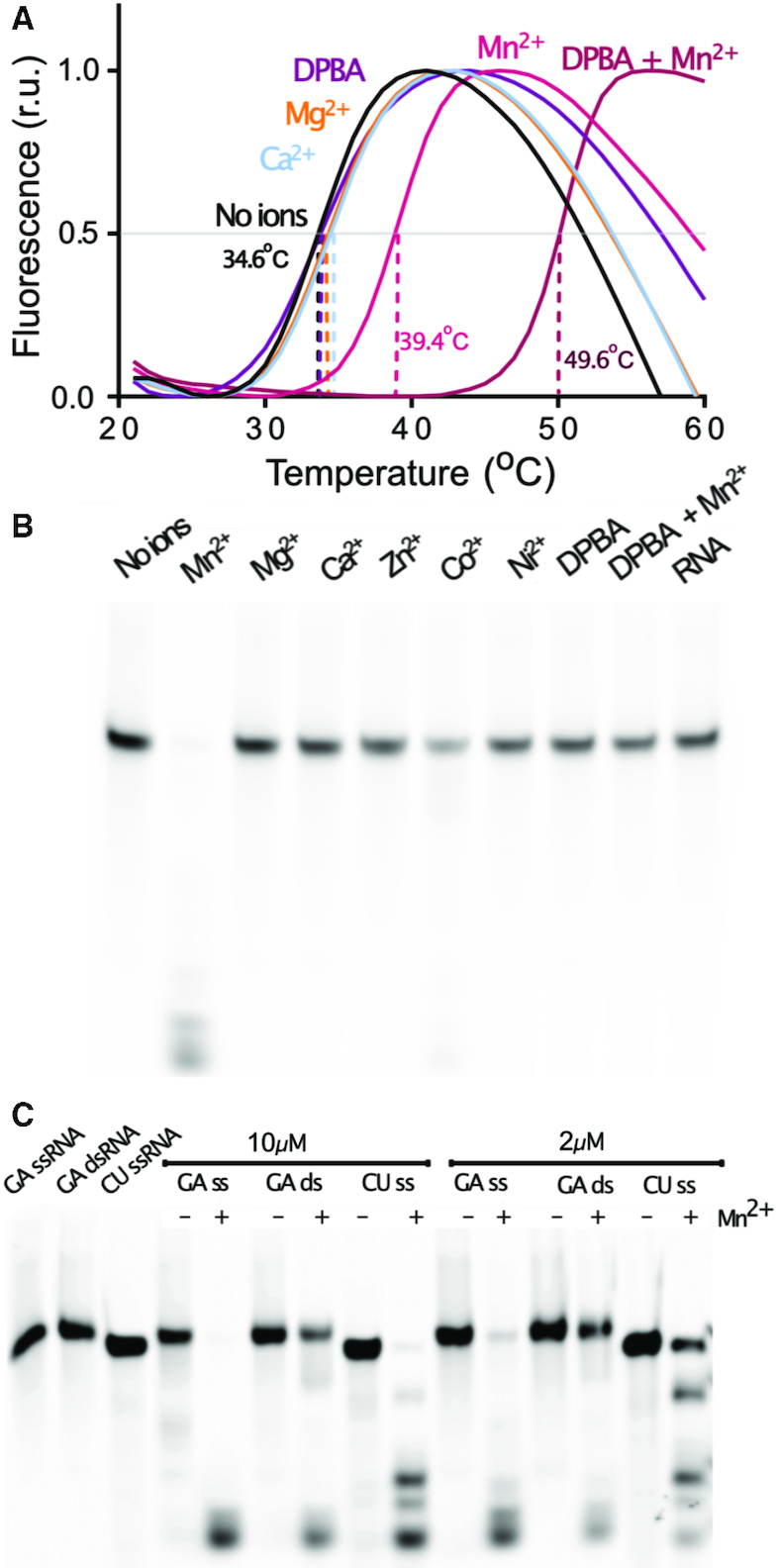
Ion stabilization and catalytic activity of the TOSV EN. (**A**) TSA experiments showing the stabilizing effect of metal ions and DPBA on the TOSV EN. The fluorescence measurements in temperature gradients are plotted for the EN in the presence of the indicated metal ions and DPBA. The dashed lines indicate the derived *T*_m_ values. The indicated *T*_m_ values are mean values from three independent experiments. (**B)** EN activity assay performed with 2 μM enzyme incubated for 1h at room temperature with GA-rich ssRNA in the presence of 2 mM of the indicated metal ions. (**C**) Mn^2+^ dependent digestion of the GA-rich and CU-rich ssRNAs and GA dsRNA at 2 and 10 μM protein concentration.

Since cap-snatching endonucleases have divalent cation dependent activity, we analysed the specificity of the TOSV EN for divalent cation binding and cation dependent activity. First we carried out thermal shift assays in the presence of different divalent cations following the denaturation curve in a temperature gradient (27) (Figure 1A). Interpretable denaturation curves were only obtained in the presence of 2 mM MnCl_2_, MgCl_2_ or CaCl_2_. Three independent experiments gave a measured melting temperature (Tm) of 34.6 ± 1.1°C in the absence of divalent cations, which did not change significantly in the presence of Mg^2+^ or Ca^2+^ ions (34.4 ± 0.3 and 34.3 ± 0.2°C respectively). In the presence of Mn^2+^, the apparent stability of the protein increased by 5°C (*T*_m_ = 39.4 ± 0.8°C). This increase in *T*_m_ is similar to that previously reported for the Hantaan virus cap-snatching EN and is consistent with the *in vitro* Mn^2+^ binding preference of other cap-snatching ENs (25). Another feature of cap-snatching ENs is that their activity can be inhibited by di-ketoacids, that bind to the active site in an ion dependent manner (e.g. DPBA (15,18,25)). When 0.5 mM of DPBA inhibitor was added, we detected a further increase in thermal stability of 10°C that was dependent on the presence of Mn^2+^ (*T*_m_ = 49.6 ± 0.2°C). This stabilization induced by Mn^2+^ and DPBA is similar to that observed for all His+ and His– cap-snatching ENs described to date with the notable exception of the inactive THOV virus EN, that lacks any divalent cation binding (26).

In parallel, we tested the endonuclease activity under the same conditions with a 5′ FAM fluorescently labelled GA-rich 25mer RNA, also including Zn^2+^, Co^2+^ and Ni^2+^ metal ions in the assays (Figure 1B). The EN shows robust activity only in presence of Mn^2+^ and detectable but very weak activity with Co^2+^. No activity or RNA degradation was detected in the control reactions in the absence of protein or metal ions. When the DPBA inhibitor was included at 0.5 mM concentration in the reaction with 2 mM Mn^2+^, the reaction was completely inhibited. As expected, the control reaction with only DPBA showed no activity. Thus, the TOSV EN behaves as an active Mn^2+^ dependent EN that can be inhibited by DPBA, similarly to other previously described His+ cap-snatching ENs.

In order to characterize the RNA substrate specificity of the TOSV EN we compared the endonuclease activity using two 24 and 25 nucleotides long, single stranded RNA (ssRNA) substrates: one purine rich (GA-rich) and another pyrimidine rich (CU-rich). Figure 1C shows the reactions at two different protein concentrations. The digestion of the CU-rich was consistently less efficient than the GA-rich digestion, indicating a preference of the EN for purine-rich RNA substrates and thus strongly suggesting that specific interactions occur between the RNA bases and EN during substrate recognition. We also noticed that the EN was not able to completely degrade the RNA, thus digestion products obtained from GA or CU RNA cleavage were analysed by electrophoresis in high-resolution urea/acrylamide gels (Supplementary Figure S1A). In both cases the digestion fragments were never shorter than five nucleotides, suggesting that the minimal length for a cleavable RNA substrate is six nucleotides. Certain cleavage intermediates were accumulated during the reaction but no sequence specificity could be assigned to the cleavage patterns obtained (Supplementary Figure S1B). The DPBA inhibitor at 0.5 mM completely inhibited the endonuclease activity. When the GA-rich ssRNA was annealed with a complementary RNA to produce dsRNA, the reaction efficiency dramatically dropped, indicative of the TOSV EN preference for ssRNAs.

In order to better assess the efficiency of TOSV EN activity we performed real time endonuclease activity measurements in a FRET based assay. In this assay the RNA substrate is labelled with a fluorophore at the 5′ end (6-FAM) and a quencher at the 3′ end (BHQ1) resulting in an increase in fluorescence emission upon cleavage of the RNA. For different protein concentrations, reaction velocities were determined from the initial slope corresponding to the steady state of the reaction (Supplementary Figure S1C). The linear relationship between the calculated velocities (V = r.u/s, where ru corresponds to fluorescence relative units) at different concentrations is shown in Supplementary Figure S1D. From the slope, a processivity rate of 11.85 ru/s•μM was derived with a R^2^ correlation coefficient >0.99. This activity rate is similar to those reported for other His+ cap-snatching endonucleases (see discussion).

In summary, TOSV EN, including the first 211 amino acids of the TOSV L protein, has a robust processive Mn^2+^ dependent nuclease activity for ssRNA, that is rather sequence unspecific, but with a certain preference for purine-rich RNAs. The activity can be inhibited by DPBA at 0.5 mM concentration and the reaction rate is similar to other His+ ENs. Altogether, these experiments show that TOSV EN behaves as a His+ cap-snatching EN (15,18,25).

### The crystal structure of the Toscana cap-snatching endonuclease

The crystal structure of the cap-snatching EN domain of the Toscana virus was solved using the NTos211 construct. Crystals of the apo form grew as small bars in the presence of 2 mM MnCl_2_ that diffracted to 2.4 Å. Addition of 0.5 mM of DPBA to the condition in order to stabilise the protein was required to produce crystals with SeMet derivatised protein. SeMet derivatised DPBA co-crystals grew as thick plates that diffracted to 2 Å resolution and were thus used to solve the TOSV EN structure by single anomalous diffraction (SAD). This model was subsequently used to phase data from apo crystals and crystals in an intermediary DPBA-bound state (henceforth referred to as the DPBA-off structure) via molecular replacement (see Supplementary Table S1). The protein crystallised in space group *p*2_1_2_1_2 with two molecules in the asymmetric unit (asu): hereby referred to as molA and molB.

**Figure 2.**
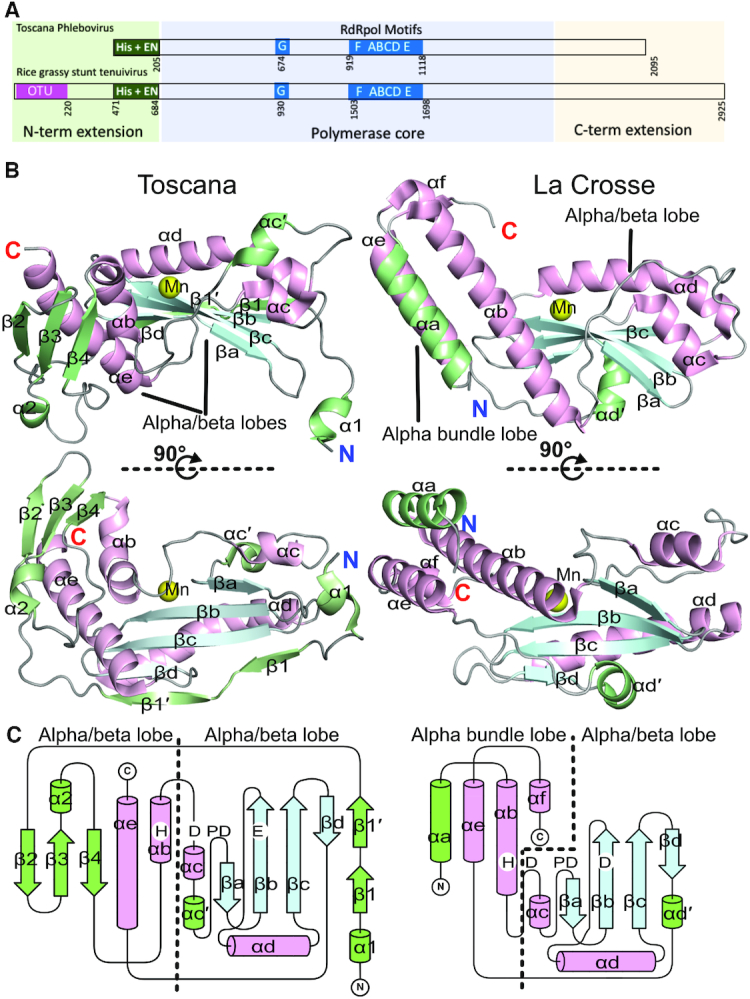
Structure of the TOSV cap-snatching EN in comparison with LACV EN. (**A**) Scheme of *Phenuiviridae* L protein domain organisation showing Toscana phlebovirus and Rice grassy stunt tenuivirus. The scheme indicates the conserved N terminal extension, the polymerase core and the C terminal extension as in Ferron *et al.* (20). The numbering of conserved OTU and EN domain boundaries and the position of the RdRp motifs are indicated. (**B)** Two orthogonal views of the apo structures of TOSV and LACV ENs represented as cartoons. The conserved alpha helices are coloured in light pink, the beta strands in light blue and loops are in grey. The strain-specific secondary structures are coloured in green. The manganese ions in the active sites are shown as yellow spheres. The nomenclature of secondary structures is numeric for the strain specific secondary structures and alphabetic for conserved secondary structures in agreement with Reguera *et al.* (18,25). The alpha/beta and the alpha bundle lobes are indicated. (**C**) Folding scheme of the two ENs. Cylinders represent alpha helices, arrows represent beta strands and loops are drawn by lines. The colouring is the same as in panel B. The position of the catalytic residues is indicated (H-D-PD-D/E). It is possible to see how the N terminus of TOSV has changed its folding in comparison with LACV, and the other cap-snatching ENs described to date (see Supplementary Figure S2). As in panel B, the alpha/beta and the alpha bundle lobes are indicated.

**Figure 3.**
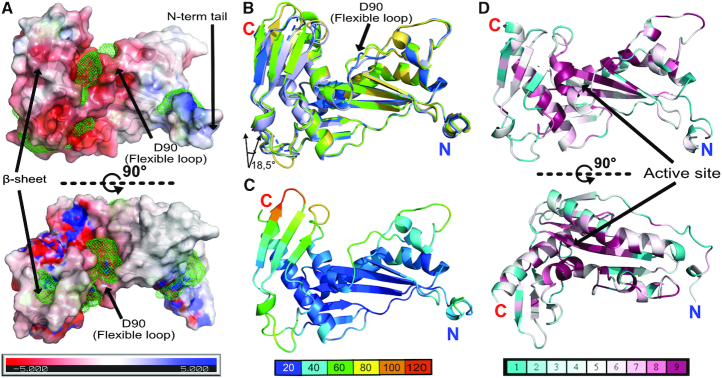
TOSV EN surface electrostatics, pockets, flexibility and conservation. (**A**) The EN surface electrostatics calculated by APBS are shown from -5 KT/e in red to +5 KT/e in blue, coloured as indicated in the bar below. The pockets shown by green mesh were calculated using the Pocasa server (http://altair.sci.hokudai.ac.jp/g6/Research/POCASA_e.html). Two negatively charged pockets appear on both sides of the beta sheet. The largest communicates with the active site, which is also negatively charged. An additional cleft with positive charge is formed between the N terminal tail and the EN body. The position of the D90 in the flexible loop is indicated. (**B**) Cartoon representation of the superposition of the two molecules from the apo (molA in green, molB in yellow) and the two from DPBA (molA in light blue, molB in navy blue) structures. Superposition shows the flexibility of the N terminal beta sheet with a maximum angular deviation of 18.5° in the hinge at the β sheet base (calculated using the DynDom server). The secondary structures are labelled as in Figure 1. (**C**) TOSV EN structure represented in cartoon and coloured according to crystallographic B-factors (indicative of flexibility), from blue (low B-factors and flexibility) to red (high B-factors and flexibility) following the bottom colour code bar. (**D**) Two orthogonal views of the TOSV EN coloured according to the amino acid conservation among phenuivirus following the bottom colour code bar from low conservation (cyan) to high conservation (purple). The figure was created using the Consurf server (http://consurf.tau.ac.il/2016/) from a Tcoffee amino acid sequence alignment including the sequences of Supplementary Figure S5 plus SFTSV, WCH/97/HN/China/2011, SFSV, Bhanja virus, Arumowot virus and Yichang insect virus.

The structure of the TOSV cap-snatching EN includes the first 205 amino acids from the L protein N terminus (Figure 2A), the remaining 6 residues at the C terminus of the construct are disordered. The structure has significant rearrangements in the domain folding compared to previously reported EN structures. To better illustrate these changes, we show the structure in comparison with the EN domain of the homologous LACV (18), the prototype of the *Orthobunyavirus* genus, in Figure 2B and C. The TOSV EN fold is organised as two alpha-beta lobes rather than the one alpha/beta and one alpha bundle lobe architecture that is conserved in LACV EN and all previously described bunyaviral cap-snatching EN structures. The first alpha-beta lobe (built from beta strands βa-d and alpha helix αd) that contains most of the catalytic residues (D-PD-ExK), is basically identical to the alpha beta lobe reported for orthobunyavirus (LACV), hantavirus and arenavirus ENs (Supplementary Figure S2). However, the TOSV EN exhibits the striking peculiarity of incorporating an additional amino terminal beta strand (β1) ending in a short alpha helix (α1) that protrudes from the protein core and forms a small positively charged pocket with the body (Figure 3A). In contrast to previously reported cap-snatching EN structures, this folding rearrangement relocates the N terminus to the opposite side of the domain and away from the C terminus that connects with the polymerase body. The second alpha beta lobe shares the alpha helices αe and part of αb with homologous ENs, but substitutes αa by a beta sheet. More subtle structural rearrangements are also observed. The alpha helix αb of TOSV, which bears the catalytic histidine, is shortened and bent in comparison to the long and straight αb of homologous ENs (Figure 2B). The long C terminal helix αe also adopts a different orientation compared to αe of homologous cap-snatching ENs. In the homologous influenza polymerase and LACV L protein structures, this helix connects with the polymerase globular core. The N terminal beta sheet (β2-4) lies across the long C terminal αe helix, and deploys long flexible loops from each side of the sheet, including a short α helix (α2) between β3 and β4. The positioning of this beta-sheet generates a large negatively charged cavity on one side of the protein, separated from the active site cavity only by the flexible loop between αb and αc that harbours the catalytic D90 (Figure 3A). As a consequence of all these modifications, the lobe is shortened, conferring a more globular shape to the EN, that still maintains the kidney-like shape found in other *Bunyavirales*.

Comparison of the asymmetric units between the apo and DPBA crystals shows that the core of Toscana EN (comprising beta strands βa-βd, alpha helices αb-αe, and the TOSV specific beta strand β1 and alpha helix α1) is quite rigid (Figure 3B). Apo molA and molB exhibit 0,316 Å RMSD for this region calculated for the Cα atoms. In this central core only a short loop, holding the catalytic residue D90, and the αc and αc′ insertion show a certain flexibility. N terminal helix α1 also exhibits low RMSD (0,376 Å for apo molA and B) between the two molecules in the asu, although it is linked to the body by a loop four residues long and forms few contacts to the protein core. This observed helix position is stabilized by crystallographic contacts with neighbouring symmetry related proteins (Supplementary Figure S3A). To assess whether the position of α1 in our structure is maintained in solution, we carried out small-angle X-ray scattering (SAXS) measurements. The scattering profile overall agrees (chi^2^ = 2.3, see Supplementary Figure S4B) with the theoretical scattering of our model in a monomeric form, strongly suggesting that in solution TOSV EN is basically folded as observed in the crystal structure. However, the α1 helix does not fit into the *ab initio* model suggesting that in solution it might have different single or multiple conformations (Supplementary Figure S4A). Indeed, comparison of the experimental scattering profile of TOSV EN with the theoretical scattering profile of TOSV EN lacking 12 amino acids from the N terminus results in a much better fit (chi^2^ = 1.5, see Supplementary Figure S4C) supporting the hypothesis that the N terminus might be rather flexible in solution. Thus, the α1 helix conformation observed in the crystal structure is likely induced by crystal contacts. The N terminal beta sheet (β2–4) also shows high flexibility. It changes orientation between the structures of molA and molB in the three structures, swinging over the long C terminal αe with a maximum rotation angle of 18.5° between molA of the apo structure and molB of the DPBA bound structure (Figure 3B) (calculated by DynDom server http://dyndom.cmp.uea.ac.uk/dyndom/ (35)). This region also has higher crystallographic B-factors for the main chain of each molecule and high RMSD (1,653 Å for apo molA and B) in comparison with the stable core (Figure 3C). This flexibility is particularly high for the β2–3, β3–4 and β4-αb loops (residues K38-G43, A54-N57 and P69-Q71 respectively); and electron density in the 2*F*_o_ – *F*_c_ maps is either weak in this region, or completely absent in the case of molB in the DPBA-off structure (Supplementary Figure S3B). Overall, the new N terminal tail and beta sheet have the highest flexibility in the TOSV EN domain.

An amino acid sequence alignment of a set of representative viruses from three genera of the *Phenuiviridae* family is presented in Supplementary Figure S5, showing the low overall sequence conservation between the ENs. However, the H-D-PD-D/E-K motif, characteristic of the His+ cap-snatching ENs, is completely conserved among all genera. In addition, when the amino acid sequence conservation is represented in the crystal structure by ConSurf (36) the residues in the domain's core are highly conserved (see Figure 3D); indicating that this structure is likely to be maintained in all cap-snatching ENs of phenuiviruses. Characteristic features for each genus can yet be found: Gokioviruses (Gouleaku and Cumuto viruses) show a five residues-insertion that elongates the β2 strand at the new alpha/beta lobe and another insertion after αd that may slightly elongate the conserved alpha/beta domain. The N termini of Gokioviruses L proteins are extended (e.g. 16 residues in Cumuto virus) with respect to phleboviruses (TOSV, Uukuniemi, RVFV and Punta Toro). These extensions are particularly striking for tenuiviruses (Rice-stripe and Rice-grassy), that include more than 500 additional residues at the N terminus (e.g. 525 extra N terminal residues for Rice-grassy virus) including an OTU domain (37).

In summary, the TOSV EN conserves the rigid catalytic core of the *Bunyavirales* cap-snatching ENs but has completely reconfigured the first N terminal 70 residues. The consequence is a relocation of the N terminus away from the polymerase core, the addition of a flexible N terminal alpha helical tail and the incorporation of a beta sheet that deploys flexible loops towards the polymerase body. This new configuration implies that phenuivirus ENs incorporate alternative structural features to establish novel interactions within the L protein or with other cellular factors.

### Structure and dynamics of the active site upon divalent ions and DPBA binding

**Figure 4.**
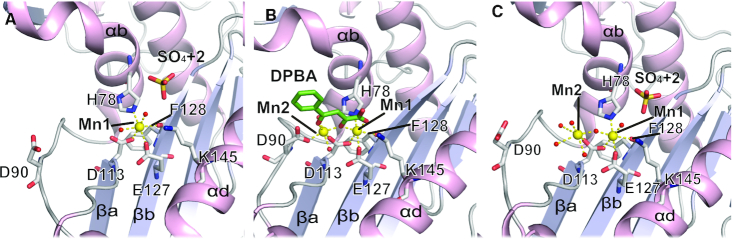
Structure and dynamics of the TOSV EN active site. (**A**) Cartoon representation of the apo structure active site. The catalytic residues and SO_4_ +2 molecule are represented as sticks and labelled. Their atoms are coloured in grey (carbon) red (oxygen), blue (nitrogen) and yellow (sulphur). Mn1 is shown as a yellow sphere and the coordinating water molecules as small red spheres. Coordination of the Mn1 is shown by dashed yellow lines. (**B**) Active site of the DPBA bound structure, represented as in panel A. The DPBA molecule is presented in sticks and colored in green. The D90 and the loops are engaged in the active site for the ion coordination, and SO_4_^2+^ disappears. (**C**) Active site of the DPBA-off structure, represented as in panel A. The D90 exits the active site, but the Mn2 remains in the active site. The SO_4_^2+^ appears again, as in the apo structure.

The active site of the TOSV EN is defined by the H-D-PD-E-K motif, and maintains the canonical configuration observed in other His+ cap-snatching ENs (see Figure 4 and Supplementary Figure S6). In the apo crystal asymmetric unit, each molecule incorporates one Mn^2+^ ion (Mn1) in the active site with real space correlation coefficients (RSCC) of 1. The Mn^2+^ ions are octahedrally coordinated by the sidechains of D113 on βa, H78 on αb, E127 on βb and the main chain carboxyl of F128 on βb, together with two water molecules, all at distances of 2.2 Å (Figure 4A). D90, residing in the flexible loop between αb and αc, is displaced from the manganese coordination site and shows high B-factors (above 94 Å^2^) compared to the low B-factors (<40 Å^2^) shown by the other highly ordered catalytic residues. The electron density for the entire flexible loop and in particular for D90 is weak but unambiguous for the main chain (Supplementary Figure S7A). K145 is located in helix αd, and forms one hydrogen bond to the water molecule that coordinates the manganese ion. The amino moiety of the side chain is identically positioned in the homologous K134 of influenza A virus, K124 of Hantaan virus, K115 of Lassa virus and K94 of LACV cap-snatching ENs. This is the equivalent position adopted by the lysine allowing the nucleophilic attack in EcoRV (K92). In EcoRV, as in LACV, the equivalent lysine is positioned in beta strand βb (Supplementary Figure S6).

The apo structure also contains seven sulphate ions bound to molA and three to molB in the asu with RSCC above 0.83. Four sulphate ions are found close to the active site (Supplementary Figure S8A). The presence of sulphate ions is common in positively charged cavities of protein crystal structures, and is a particularly well-documented phenomenon for nucleic acid binding proteins (e.g. (38,39)). Since there are no cap-snatching EN structures in complex with RNA substrate available in the PDB, we explored the possibility that some of these sulphate ions could mimic the path of the TOSV EN RNA substrate binding. Superposition of TOSV EN molA onto the homologous EcoRV EN in complex with its DNA substrate (PDB: 1EOO), identifies three sulphate ions that overlay with the phosphate groups of the DNA substrate at the –1 (SO_4_^−^), the +2 (SO_4_^2+^) and +3 (SO_4_^3+^) positions (see Supplementary Figure S8B). The distances between these three sulphate ions also match the spacing between the phosphates of an RNA molecule in the A form conformation (Supplementary Figure S8C). Supplementary Table S2 lists the contacts between the sulphate ions in the active site and the protein. SO_4_^–^ is highly coordinated by hydrogen bonds with the main chain of S111, the hydroxyl group of the Y149 sidechain and the amino moiety of K148. SO_4_^2+^, consistently present in molA and B, is stabilized by hydrogen bonds formed to T130 and T129 side and main chains respectively. It also maintains a hydrogen bond with the water that bridges the Mn1 and the K145 catalytic lysine. SO_4_^3+^ is held by hydrogen bonds with M132 main chain amino moiety and T131 sidechain. Finally, a fourth sulphate ion (SO_4_′), outside of the proposed RNA binding path, forms a hydrogen bond with K145 amino moiety. The amino acid sidechains involved in hydrogen bonds with these sulphate ions are conserved among *Phenuiviridae* ENs, with the sole exception of T131, further supporting that these sulphate ions are located in an RNA binding site (see alignment in Supplementary Figure S5).

The structure of the TOSV EN with DPBA shows the inhibitor binding through chelation of two Mn^2+^ in the active site of each molecule in the asu. The anomalous signal from the two metal ions is detected for both sites with similar intensities. The Mn^2+^ ion occupancies in the structure are of 1 and 0.72 for positions Mn1 and Mn2 respectively (Figure 4B and Supplementary Figure S7B). Mn2 is octahedrally coordinated by the central D113 and two water molecules, in addition to D90 in the flexible loop, which swings into the active site and becomes ordered relative to the apo structure. Since DPBA forms no direct contacts to the protein, the increase in protein stability measured upon DPBA binding seems to be a result of the second manganese ion (Mn2) binding to the active site, induced by the inhibitor. Upon DPBA binding the number of bound sulphate ions is also reduced relative to the apo structure, thus suggesting a lower affinity for sulphate ions. Only SO_4_^−^ is present in molB and no density is observed for SO_4_^2+^ and SO_4_^3+^ sulphate ions in molA and molB.

In order to better characterize the DPBA-binding we soaked the DPBA crystals in cryobuffer without the inhibitor, washing away the DPBA from the active site. The resolution of the diffraction improved to 1.5 Å, and the structure displays an intermediate conformation of the active site between the previous apo and DPBA bound conformations (Figure 4C and Supplementary Figure S7C). The electron density for the DPBA completely disappears but the two Mn^2+^ ions are still located in the active site and anomalous signal is detectable for both positions. Mn1 shows three-fold more anomalous signal than Mn^2+^ and the occupancies are of 1 and 0.52, respectively. The aspartic acid D90 swings out of the active site, adopting a conformation similar to that observed for the apo structure. However, the loop seems to be more disordered; electron density for the side chain of this residue is no longer observable. This leaves the octahedral coordination shell of Mn2 incomplete and coordinated only by the central D113 and two water molecules. This structure partially recovers the sulphate ions binding state of the apo structure; SO_4_^2+^ appears again in both molecules of the asu, but not SO_4_^−^ or SO_4_^3+^. Since the *apo* structure, obtained with the same cryo-buffer than the DPBA-off structure, has only Mn1 in the active sites, this two Mn^2+^ bound intermediate conformation indicates that the di-ketoacid inhibitor release and the exit of D90 from the active site is faster than the Mn2 metal ion release.

Altogether these three structures show that the TOSV EN has the active site conformation and behaviour of a His+ cap-snatching EN, despite the major structural rearrangements observed in the folding of the N terminal lobe. The apo structure provides evidence for the role of highly conserved residues for substrate binding and, by comparing it with the DPBA and DPBA-off structures, we can better understand the dynamics for substrate binding and product release. The movement of D90 towards the active site is the main feature underlying these dynamics, and as previously suggested for the homologous LACV EN, may play a regulatory role for the EN activity (18).

### Role of the active site, sulphate ions binding and N terminal residues on the Toscana EN endonuclease activity and ion induced stability

**Figure 5.**
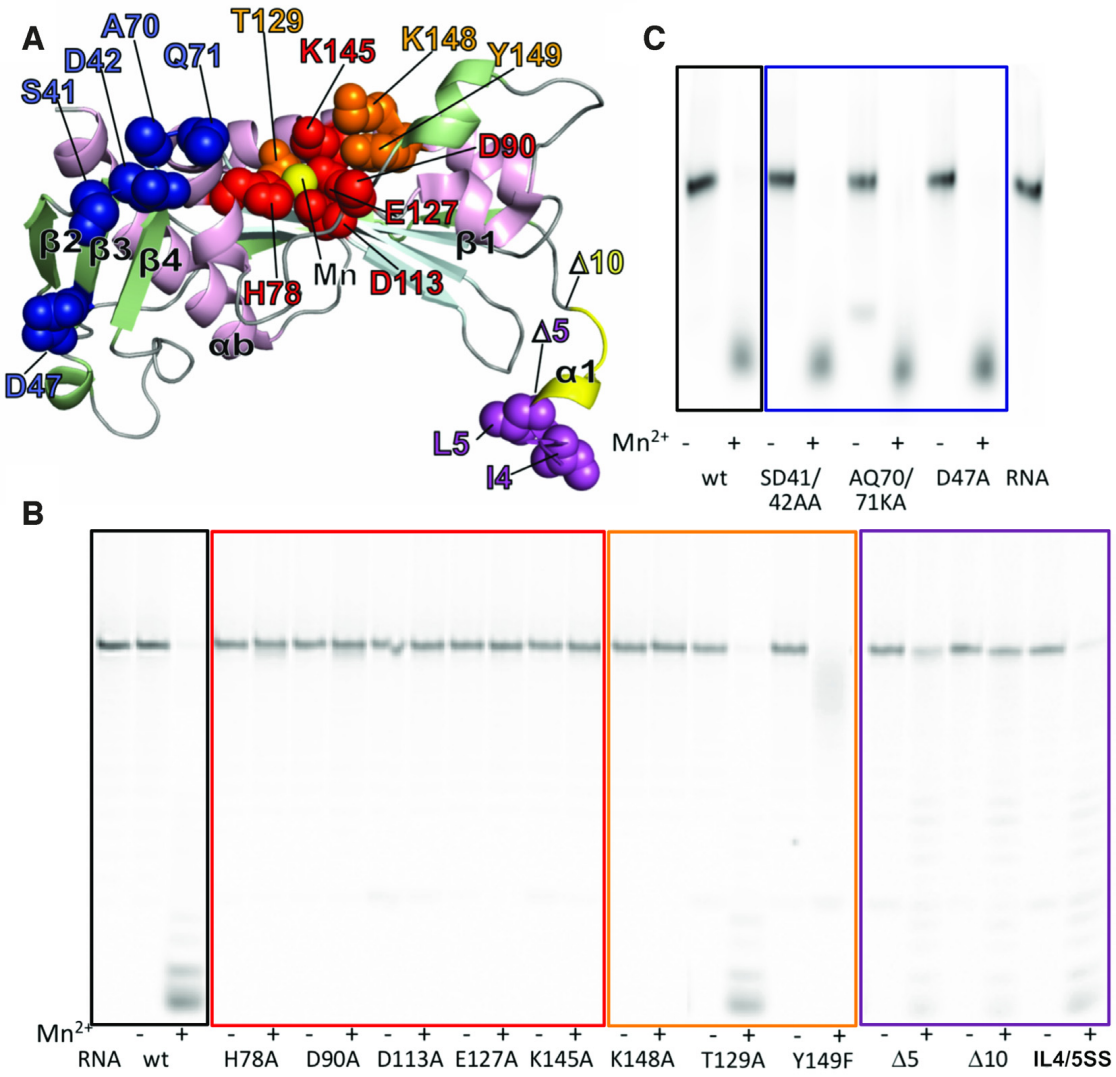
Endonuclease activity of TOSV EN mutants. (**A**) Mutations mapped onto the structure of TOSV EN, represented as in Figure 2. Mutations in active site residues are represented as red spheres, sulphate ions binding residues in orange, beta-sheet loops in blue and mutations and deletions in the N terminal region are indicated in purple (between residues 1 to 5 (Δ5)) and yellow (between 6 to 10 (Δ10)). (**B**) Ion dependent EN activity detected for proteins with mutations in the active site (red square), sulphate ions binding residues (orange square) and N terminus tail (purple square) with the wt as control. We show a representative experiment out of three independent experiments. (**C**) The same for the N terminal beta sheet mutants (blue square). All assays were performed with 0.5 μM GA ssRNA and in the presence or absence of 2 mM MnCl_2_. Some background residual activity, independent of metal ions, appears sometimes in the assays. It seems associated with contaminations of samples rather than TOSV EN activity. For example, in this panel, this activity is most pronounced for the AQ70/71KA double mutant, but can also be noticed in panel B for several mutants with lower intensity. As in panel B, we show a representative experiment out of three independent experiments.

Based on our structural data we carried out site directed mutagenesis of active site residues (H78A, D90A, D113A, E127A and K145A), residues involved in sulphate ions interactions (K148A, T129A, Y149F), residues located in the N terminal beta sheet and exposed loops (SD41/42AA, AQ70/71KA, D47A) as well as deletions and site mutations of the N terminal α1 tail (Δ5, Δ10, IL4/5SS) (Figure 5A). We analyzed the effect of each mutation on protein stability, metal ion and DPBA binding, and catalytic activity. The results are presented in Figures 5B and C and Table 1.

**Table 1.** Endonuclease activity, thermal stability and Mn^2+^ ion and DPBA induced stability of TOSV EN mutants versus wt. The mutants are coloured following Figure 5A color code. ^1^Mutant EN activity is classified relative to the wt protein as entirely active (++), partially active (+), barely active (±) and inactive (−). The classification reflects results from at least three independent EN activity experiments (representative experiment shown in Figure 5B and C). ^2^The *T*_m_ values calculated from TSA experiments are shown for wt and mutant proteins in the absence of ions, the presence of 2 mM MnCl_2_ and the presence of 2 mM MnCl_2_ and 0.5 mM DPBA. Standard deviations are from three independent experiments, where readings were performed in triplicate for each experiment

		Thermal stability^2^		
	EN activity^1^	No ions	Mn^2+^	Mn^2+^+DPBA
**WT**	**+++**	34.6 ± 1.1	39.4 ± 0.8	49.6 ± 0.2
**D113A**	**−**	31.5 ± 0.8	31.6 ± 0.5	32.1 ± 1.0
**E127A**	**−**	35.5 ± 2.7	36.1 ± 0.3	37.1 ± 0.2
**H78A**	**−**	38.0 ± 0.7	37.6 ± 0.9	37.3 ± 2.4
**D90A**	**−**	35.9 ± 0.5	40.1 ± 0.5	49.3 ± 0.1
**K145A**	**−**	35.9 ± 0.3	43.8 ± 0.3	51.4 ± 0.1
**K148A**	**−**	35.7 ± 0.6	43.7 ± 0.5	52.2 ± 0.1
**Y149F**	**−/+**	31.3 ± 0.9	38.2 ± 0.6	49.9 ± 0.1
**T129A**	**+++**	34.1 ± 0.2	39.3 ± 0.0	50.4 ± 0.0
**D47R**	**+++**	32.4 ± 0.2	37.5 ± 0.3	49.5 ± 0.1
**SD41/42AA**	**+++**	32.2 ± 0.7	37.4 ± 0.7	48.6 ± 0.1
**AQ70K/71KA**	**+++**	36.7 ± 1.2	40.9 ± 1.2	50.7 ± 0.1
**IL4/5SS**	**++**	33.9 ± 0.0	38.7 ±- 0.0	46.7 ± 0.2
**Δ5**	**+**	31.6 ± 0.7	34.5 ± 0.8	45.3 ± 0.1
**Δ10**	**−/+**	35.0 ± 1.3	36.4 ± 0.8	45.2 ± 0.4

Mutations in the active site residues involved in Mn^2+^ coordination (D113, E127 and H78) result in a lack of ion and DPBA binding and Mn^2+^ dependent EN activity. Mutation of D90 to alanine did not affect the stabilization of the protein by Mn^2+^ ions. This suggests that the 5°C of Tm stabilization observed for the wild-type (wt) protein is due mainly to the binding of a Mn1, as D90 only participates in coordination of Mn2. This mutant also shows a Tm increase in the presence of DPBA, suggesting that loss of the D90 hydrogen bond to Mn2 does not prevent binding of the DPBA inhibitor via the two Mn^2+^ ions. This is consistent with the DPBA-off structure where the intermediary DPBA release state has both Mn^2+^ ions still bound even though D90 is disengaged from the active site. Nevertheless, the mutation renders the protein inactive, indicating that the D90 coordination to Mn2 is important for the EN activity. Mutation of the catalytic lysine K145A did not affect protein stability. However, the stabilisation increase induced by Mn^2+^ ions and DPBA appear slightly higher relative to the wt protein. Nonetheless, the mutation abolished the endonuclease activity of the protein. Thus, only H74, D113 and E127 are essential for metal ion binding, but all catalytic residues are essential for enzymatic activity. The functional relevance of these residues is further reflected by their complete conservation among *Phenuiviridae* cap-snatching ENs.

Mutation of residues involved in sulphate ions binding Y149F and T129A did not affect the stabilisation of the protein by Mn^2+^ and DPBA. Only K148A, analogously to K145A, shows a higher stability increase induced by Mn^2+^ and DPBA than the wt protein. Nonetheless, the K148A mutation, removing a salt bridge with SO_4_ -1, results in loss of EN activity *in vitro*. Y149F and T129A, mutations that remove one hydrogen bond to either SO_4_^–^ or SO_4_^2+^, show an incomplete degradation of the RNA substrate or almost no effect in comparison with the wt, respectively. This activity drop supports the RNA binding role of these residues as suggested by their interactions with sulphate ions in the apo structure.

Additional mutations in the TOSV EN N terminal tail were carried out to investigate the possible role in protein stability and catalysis. Deletions of the first 5 and 10 amino acids (Δ5 and Δ10) as well as the substitution of two hydrophobic residues, I4 and L5, by serines (IL4/5SS) were analysed. These two residues are conservatively mutated among phlebovirus and exposed to the solvent in the structure. These mutations slightly reduced the stability of the protein by 2–3°C, like the D113A and Y149F mutants, and showed the same pattern of stabilization by Mn^2+^ and DPBA observed for the wt (Supplementary Figure S9 and Table 1). Surprisingly, all of the mutations affected the Mn^2+^ dependent EN activity of the enzyme, resulting in almost total (Δ10) to partial (IL4-5SS) loss of activity. Since the N terminal helix is distant from the active site, the helix could have an indirect role in substrate binding, which could explain the TOSV EN need for long RNA substrates for cleavage.

Finally, we tested mutations in exposed regions of the N terminal beta sheet to see whether they influenced the activity or stability of the EN domain. Residues were mutated in the β2–β3 loop (SD41/42AA), the β4–αb loop (AQ70/71KA) and in the external surface of the sheet (D47R). These regions could be interacting with the polymerase core of the full-length L protein (see Figures 2 and 7). As expected, these mutations did not significantly affect the stability, Mn^2+^ and DPBA binding or catalytic activity of TOSV EN. Thus, unlike the residues in the α1 helix, these residues are not involved in any aspect of the TOSV EN enzymatic activity.

### Role of the TOSV EN active site, sulphate ions binding and N terminal residues on transcription and replication

In order to understand the biological relevance of the new *Phenuiviridae* EN structural features, we further investigated the role of residues involved in catalysis, substrate binding and putative interactions with the polymerase core in the context of the full-length L protein. Since there are no available minireplicon systems for TOSV, we performed the mutations in the homologous RVFV L protein based on sequence alignments (Supplementary Figure S5). We analysed genome replication and transcription activities of wt and mutant L proteins by using a recently developed RVFV-based minireplicon system (32) expressing *Renilla* luciferase. Overall transcription and genome replication activity of wt and mutant L proteins was assessed by measuring luciferase activity. In addition, we analysed the production of viral antigenomic and messenger RNAs by Northern blot allowing for the discrimination of effects on viral genome replication and transcription by specific mutations (for experimental details see methods).

**Figure 6.**
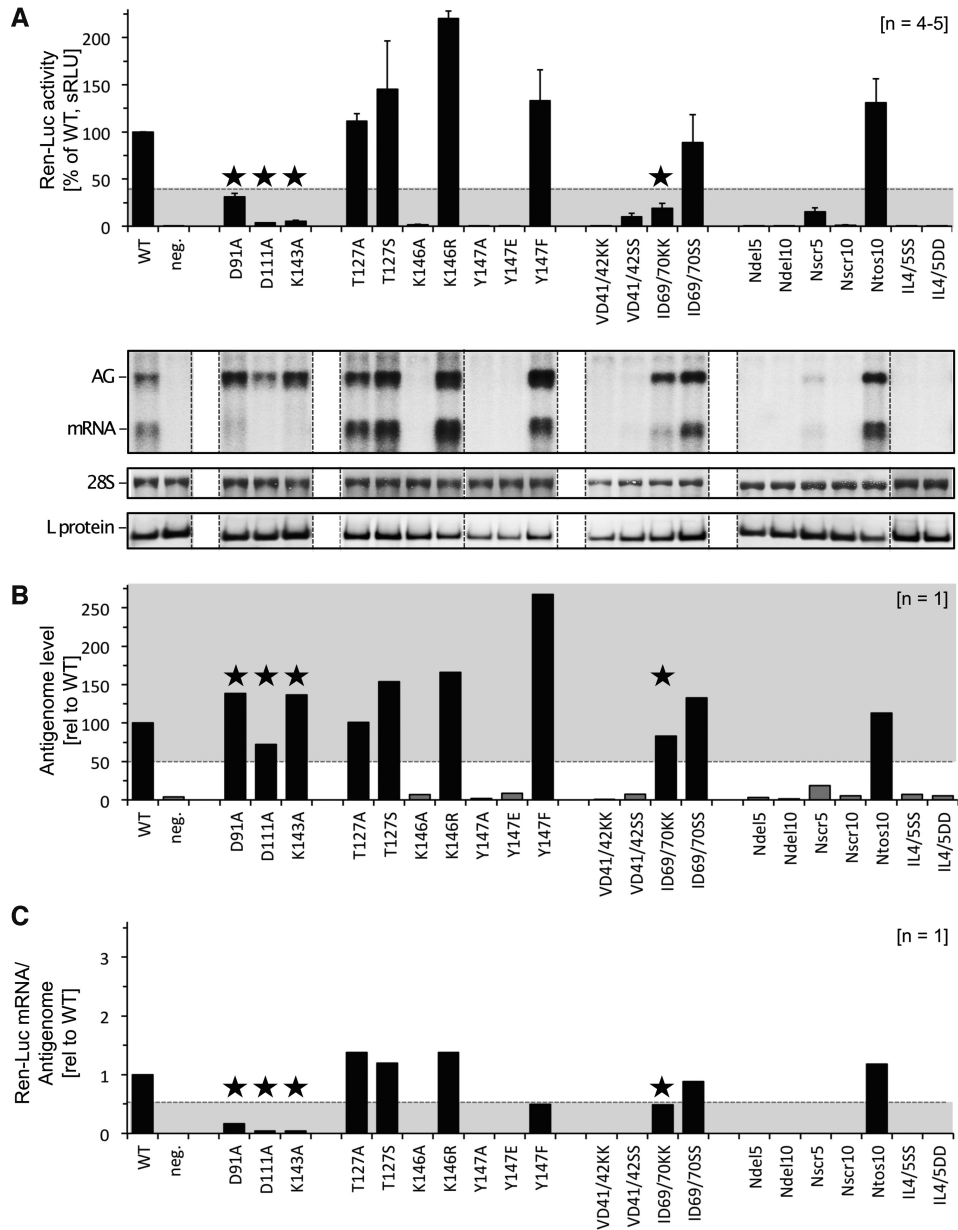
Functional investigation of RVFV L protein mutants using the RVFV ambisense minigenome system. (**A**) Transcriptional activity of RVFV L protein mutants was measured via Renilla Luciferase (Ren-Luc) reporter gene expression. The Ren-Luc activity was normalized to the firefly activity (transfection control) resulting in standardized relative light units (sRLU). The bar graph shows mean and standard deviation as a percentage of the wt activity calculated from 4–5 independent transfection experiments [*n* = 4–5]. A defective L protein with a mutation in the catalytic site of the RdRp served as a negative control (neg.). Among other criteria, mutants with a selective defect in viral transcription are expected to show signals <40% of wt activity (range is indicated by grey background). Synthesis of Ren-Luc mRNA and antigenome was evaluated by northern blotting and the northern blot signals were quantified using ImageJ2 software. All lanes displayed are from the same northern blot membrane (and same autoradiogram), dotted lines indicate cutting of the lanes for presentation reasons. The original blot is presented in Supplementary Figure S10. The data are also presented numerically in Supplementary Table S3. The methylene blue-stained 28S rRNA is shown as a marker for gel loading and RNA transfer. Immunoblot analysis of FLAG-tagged L protein mutants is also shown (L protein). Columns of mutants with a selective defect in viral transcription are marked with an asterisk. For experimental details see methods section. For quantitative analysis and definition of selective transcription defects see Supplementary Table S3. (**B**) This column chart shows the antigenome levels determined by northern blot quantification relative to the wt level. L protein mutants with a selective transcription defect are expected to show wt like antigenome levels (≥50%, indicated by grey background). Columns of mutants with a selective defect in viral transcription (as deduced from panel C) are marked with an asterisk. (**C**) This column chart displays the mRNA-to-antigenome signal ratio, reflecting viral transcription versus replication, relative to the wt as calculated from the quantified northern blot signals. The expected range of signal ratio for mutants with a selective defect in mRNA production is indicated by grey background (≤0.50). Columns of mutants with a selective defect in viral transcription are marked with an asterisk. Those mutants with no detectable antigenome yields (see panel B), thus deficient in replication, are not shown.

The mutation of RVFV EN catalytic residues D111, K143 and D91 (homologous to TOSV EN D113, K145 and D90) to alanine resulted in a significant loss of luciferase activity and a decrease in mRNA production (Figure 6A and Supplementary Table S3). Indeed, although the levels of antigenome production are similar to the wt (Figure 6B), the ratio of mRNA/antigenome is significantly low for these mutants (Figure 6C). Thus, these mutations are selectively affecting viral transcription but not genome replication. These results agree with the complete lack of activity of TOSV EN mutants D113A and K145A *in vitro* (compare Table 1 and Supplementary Table S3). In the case of the D91A mutation, it is still able to produce low amounts of mRNA and retains some luciferase activity in the minireplicon system. This is in line with the semi-essential role of TOSV D90 for the Mn-coordination as concluded from the DPBA-off structure and supports the hypothesis that the EN is capable of residual catalysis in the context of the full-length L protein, even when D90 is disengaged from the active site.

Mutations of RVFV L protein residues involved in sulphate ions interactions, T127, K146 and Y147 (homologous to TOSV T129, K148 and Y149), were performed to assess the relevance of proposed RNA binding residues for the L protein function. When K146 is mutated to alanine the luciferase activity, antigenome and mRNA production are completely abrogated (as observed for the K148A mutant in *in vitro* EN assays). The mutation does not obviously affect the full-length L protein expression or EN recombinant expression and thermal stability. Thus, this residue seems to be essential to the function of the L protein for both transcription and replication. When mutated to arginine (K146R) the luciferase activity increased by two-fold; boosted by an increase of antigenomic and mRNA levels. The positive charge on this residue is thus essential for transcription and replication activities of the L protein. The residue Y147 was mutated to alanine, glutamic acid and phenylalanine. The first two mutations that replace the aromatic side chain by aliphatic or negatively charged side chains result in a loss of any L protein activity and also seem to cause a reduction of expression, suggesting that an aromatic residue is essential at this position for substrate binding or protein folding and/or protein stability. The conservative mutation Y147F, retaining the aromatic character of the side chain, shows luciferase activity similar to the wt, although the ratio of mRNA to antigenomic RNA is reduced in relation to the wt protein, which seems to originate from an increase in genome replication (antigenome yields). Coherently, this mutation removes the hydroxyl group responsible for SO_4_^–^ coordination and has reduced endonuclease activity in *vitro*. The T127 mutant has a similar phenotype to the wt protein when mutated to A or to S, thus its interaction with the RNA substrate does not seem to be critical for transcriptional activity, correlating with the observed retention of EN activity *in vitro*.

**Figure 7.**
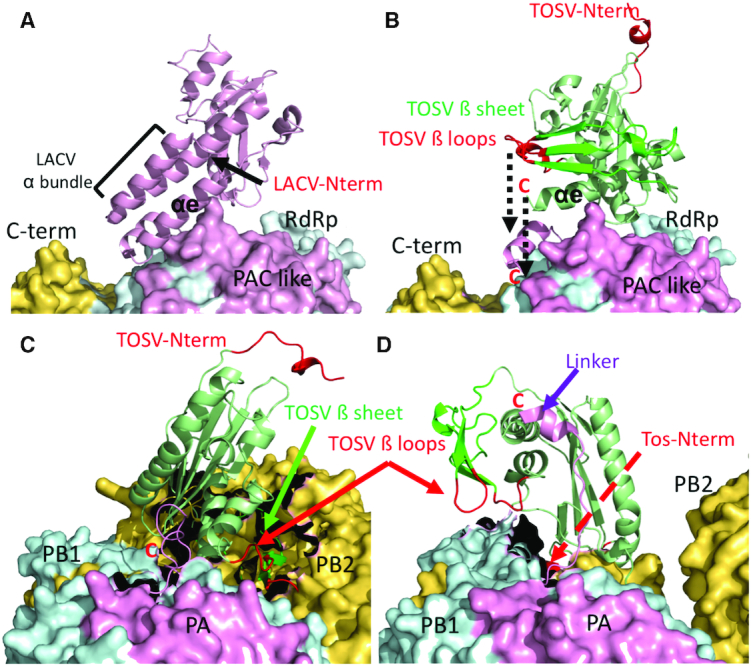
Superposition of the TOSV EN structure on the EN domains of the available structures of LACV and influenza polymerases showing the positions of the TOSV EN N terminal beta sheet and helix α1 with respect to the polymerase body. (**A**) Surface representation of the LACV L protein structure (PDB: 5AMR). The EN domain is represented in pink cartoon indicating the alpha bundle lobe. The surface of the L protein body is coloured in pink for the PAC like domain, in cyan for the RdRp region and in yellow for the fragment of the C terminal region. (**B**) The same as in A but without the LACV EN that is replaced by the superposed TOSV EN. The dashed black arrow indicates the gap between the C terminus of the TOSV EN and the connection with the L protein body at the end of αe. This approximation to the L protein body would place the TOSV beta-loops (in red) closer to the L protein surface between the RdRp and C terminal regions (dashed line arrow). The N terminus appears opposed to the L protein body. (**C**) Same representation of the TOSV EN superposed in the EN of the influenza C polymerase structure (PDB: 5D98). The PA, PB1 and PB2 subunits are coloured following the same pattern as for homologous LACV L protein; pink, cyan and yellow respectively. The beta sheet and beta loops appear in close contact with the PB1-PB2 interface (indicated by green and red arrows respectively), the black zones braking the surfaces are the regions contacting the influenza EN in the structure. (**D**), The same representation of the TOSV EN superposition on the structure of the FluB polymerase (PDB: 4WSA). In this case the linker joining the EN with the polymerase body is extended (drawn as pink coloured cartoon and highlighted by a purple arrow). The N terminus of the linker is close to the indicated C terminus of the superposed TOSV EN. In this case the beta loops are extended towards a different region of the PB1–PB2 interface. The TOSV EN N terminal tail is deployed behind the polymerase body (indicated by a red dashed arrow).

We explored the role of exposed residues located in the N terminal beta strand loops oriented towards the C terminus (the L protein polymerase core) on L protein function. The β2–3 loop residues V41–D42 (homologous to TOSV S41–D42), and β4–αb I69-D70 (homologous to TOSV A70–Q71), were substituted by lysines or serines in order to disrupt any putative contacts with the L protein body (Figure 7). Mutations in the β2–3 loop resulted in a defect of both genome replication and transcription activity, whilst equivalent mutations in the isolated TOSV EN domain i*n vitro* had no effect on protein stability or activity. This suggests that these residues are essential for genome replication and transcriptional processes beyond the endonuclease activity in the L protein. Double mutations of I69-D70 in the β4-αb loop resulted in a selective reduction of transcription activity when substituted to lysines but had no effect when substituted by serines. Since mutations in TOSV EN equivalent loops did not affect the EN activity *in vitro*, the observed reduction in transcription in the minireplicon system is likely caused by a steric effect of the two long and flexible charged sidechains in the loop, possibly hindering cross-talk between the EN domain and the polymerase core of the L protein.

Finally, several mutations of the N terminal tail were introduced in the L protein; the first 5 and 10 amino acid residues were either deleted (Ndel5 and 10) or substituted by a scrambled (same amino acid composition but changed positions) amino acid sequence (Nscr5 and 10). The residues I4 and L5, forming a conserved hydrophobic patch, were also mutated to serines or aspartic acids. All mutations resulted in a complete loss of antigenomic and mRNA production leading to complete absence of luciferase activity, with the exception of Nscr5 that retained some very low luciferase activity. Expression tests verified that the mutant full-length L proteins are produced and as stable in cells as the wt L protein. Since we also observed a decreased EN activity of the TOSV domain with deletions of the first 5 and 10 residues *in vitro*, we conclude that the N terminal tail is essential for genome replication and transcription. Surprisingly, the equivalent mutation IL-4/5-SS in TOSV EN did not abrogate EN activity *in vitro* whereas in the minireplicon system both genome replication and transcription were absent. This points towards a specific and important role of these conserved residues in the context of the full-length L protein. When the first 10 amino acids were substituted by the TOSV sequence the L protein behaved like the wt, retaining full transcriptional and replication activities. This demonstrates that the essential role of the N terminal tail for L protein function is conserved among phleboviruses and reinforces the transferability of our conclusions between TOSV and RVFV L proteins. The same mutations were further tested in a virus-like particles (VLP)-based minigenome reporter assay including plasmids expressing viral glycoproteins. These experiments confirmed the luciferase activity yields previously observed for wt and mutant L proteins, and in addition we could detect significant VLP reporter activity over the background in VLP-infected indicator cells only for the wt, Ntos10 and a transcription deficient control mutant D111A (22) (for experimental details see Supplementary Methods). This confirms that the N terminal mutations are affecting L protein activity rather than blocking their packing into VLPs (Supplementary Figure S11).

## DISCUSSION

### Implications of the TOSV EN structure for understanding L protein function and evolution

The cap-snatching ENs are key players in sNSV transcription initiation. The structures available for influenza full-length polymerase and the LACV C terminally truncated L protein show that the bunyavirus ENs are flexibly linked to the central cage-like core of the RdRp. This is most likely true for all sNSVs (6,8). The mechanism of action of the ENs in the context of the full-length polymerases is better understood through several structural studies reported for the influenza virus polymerase. The structures of influenza A, B and C strains show different conformations of mobile N and C terminal extensions, including the cap-snatching EN and cap binding domains respectively. The polymerase conformations seem to be determined by the binding of viral 3′ and/or 5′ genomic extremities (5,40,41). In addition, host factors can influence the activity of the EN. For instance, influenza polymerase is known to interact with the C terminal domain of RNA polymerase II in order to promote the capture of nascent capped cellular RNAs, substrates of the EN for cap-snatching (42). This interaction is proposed to induce the transcriptionally active conformation of the polymerase (43).

To date, the information elucidated for influenza polymerase is only partially translatable to *Bunyavirales*. The absence of full-length structures for L proteins prevents the generalization of the influenza cap-snatching mechanism. Indeed, the peculiarities of arenavirus and THOV cap-snatching mentioned in the introduction suggest that there are certainly some differences among sNSVs cap-snatching. Nevertheless, these studies illustrate how parameters such as EN substrate binding or sequence cleavage specificity need to consider other polymerase regions that certainly influence the EN activity (41). Furthermore, host factors also play an important role in bunyaviral transcription since the transcription elongation is dependent on the viral mRNA translation by the cellular machinery (44). In this context, the structure of the TOSV EN reveals significant structural changes with respect to previously reported cap-snatching ENs that could certainly have consequences on the cap-snatching mechanism. The N terminal β2-4 beta sheet replaces the helical bundle of the other *Bunyavirales* ENs reported to date. By superposing the TOSV EN onto the structure of the C terminally truncated LACV polymerase we see how the shorter TOSV beta lobe would bring the EN active site closer to the polymerase body (Figures 7A and B). The β2–3 loop and the β4–αb loop would be in close proximity with the polymerase, perhaps maintaining important contacts that regulate its function. The observed high flexibility of the β loops in the crystal structures could also be stabilized by direct interactions with the polymerase body. Thus, the implications of the N terminal re-configuration shown by the TOSV EN could reflect differences in the mechanisms of cap-snatching regulation by changing the crosstalk between the EN and the polymerase body. Indeed, in this study we demonstrate that whilst these regions are not crucial for *in vitro* EN activity, they are crucial for transcription and replication in cellular assays performed with the full-length L protein, highlighting the essential role of the interactions of the EN with the rest of the L protein.

Another striking feature of the TOSV EN structure is the relocation of the N terminus and the incorporation of an α helix (α1). This relocation would allow the insertion of large protein fragments in the N terminus for the gain of new functions. With the N terminus close to the polymerase body (as in all EN structures previously reported), insertions would sterically hinder the manoeuvre movements of the EN domain. This can be illustrated by the superposition of the TOSV EN on the different structures of influenza full-length polymerases with different relocations of the EN domain (Figure 7C and D). Indeed, note that all previously characterized ENs belong to families with a very stable L protein size (20). In contrast, phenuiviruses present a large variability in L protein sizes. Thus, we propose that this relocation is a structural solution to allow the extension of the polymerase without burdening the conformational rearrangements of the EN required during the transcription process. We can find similar extensions in *Nairoviridae* and *Tospoviridae* families and these have important functional implications such as the incorporation of an OTU domain by nairovirus, also present in tenuivirus L proteins, that is responsible for inhibiting the ubiquitin- and ISG15-dependent antiviral pathways (45,46). In the case of TOSV EN, the N terminal tail seems to be playing a role in the EN activity that could be related to substrate binding. Interestingly, the minireplicon and VLP studies indicate that the tail is also essential for viral replication and mutation of the hydrophobic residues I4 and L5 to polar residues is sufficient to completely abolish the function of the L protein. The N terminus of the L protein could be involved in cross talk between the EN and the C terminus of the polymerase, or perhaps in the interaction with necessary host factors for viral RNA synthesis. These questions remain to be addressed. In any case, whatever the precise function of this tail, it is conserved among phleboviruses, as indicated by the retention of wt activity shown by the RVFV L protein with the TOSV tail in the minireplicon assays.

### Implications of TOSV EN active site conservation and behaviour for design of broad-spectrum inhibitors

Despite the differences in folding that exist among cap-snatching endonucleases, the similarities in their active sites give hope for the possible development of broad-spectrum inhibitors (see Supplementary Figure S6). A common feature of all reported structures are two central acidic residues coordinating the Mn1 site and one catalytic lysine in equivalent positions (e.g. TOSV D113, E127 and K145). This is also true for EcoRV, and is a general feature of ENs belonging to the PD-D/ExK superfamily suggesting a certain conservation of their catalytic mechanism. Mutagenesis studies performed in this paper and with other homologous ENs demonstrate that these three residues are essential for activity both in His+ and His- ENs.

A third acidic residue is present in all cap-snatching ENs in the loop adjacent to the active site, equivalent to TOSV EN D90. The role of this residue changes between His+ and His– ENs. In arenavirus (His- ENs) the equivalent residue (Lassa D66 or LCMV D65) has never been found to coordinate the second Mn2 ion in any reported structure. This is associated to the non-canonical metal ion binding shown by His- ENs (see PDB: 5J1P in Supplementary Figure S6). Nonetheless, even upon induction of canonical Mn^2+^ ion binding by di-ketoacid inhibitors (47) (in PDB:5T2T for LCMV), D65 does not coordinate Mn2 in LCMV EN. Minireplicon studies showed that this residue (Lassa D66) is dispensable for viral transcription (25). Conversely for His+ ENs, the acidic residues homologous to TOSV D90 tested to date have been shown important for EN activity *in vitro* and for L protein activity in minireplicon assays (18,25,48,49). In LACV and TOSV the aspartic residues D52 and D90 appear disengaged from the active site and need DPBA binding (or possibly substrate binding) to approach and coordinate to Mn2 (18). The homologous Hantaan virus glutamic acid residue E54 and influenza E80 can coordinate the metal ion in the Mn2 position in the absence of inhibitors at similar MnCl_2_ concentrations, (PDB: 5IZE and 4AVQ respectively) (15,25). Thus, it is possible that longer acidic side chains (E instead of D) favour the coordination to the metal ion in the Mn2 position. These differences among His+ *Bunyavirales* endonucleases don’t have a dramatic impact on the ENs activity rates since the Mn^2+^ dependent EN activity rates of TOSV EN are similar to other His+ ENs: 11,85 ru/s•μM versus 14,38 ru/s•μM for Hantaan, 69,45 ru/s•μM for LACV and 74.88 ru/s•μM for Influenza virus (His+ cap-snatching ENs). The big difference arises with the changes observed in Lassa arenavirus (His- cap-snatching ENs) that have residual activity (0,08 ru/s•μM) (25).

The flexibility in the D90 loop also has important implications for inhibitor design. The DPBA-off structure reported here indicates that the DPBA inhibitor release results in an intermediate state where the EN still maintains the two metal ions in the active site with the D90 loop disengaged. Thus, the binding of the second Mn^2+^ ion and engagement of the D90 loop are two related but separate events, which could explain the residual transcriptional activity detected for the D91A mutant in the RVFV L protein. This mechanism of inhibitor release thus implies that di-ketoacid inhibitors with low affinity may be able to induce the active state of the endonuclease by stabilizing the two ions in the active configuration. As a consequence, there is a risk that these low affinity compounds could actually enhance viral transcription. Considering the structure of LCMV EN in complex with a L742001 di-ketoacid compound, this also seems valid for His– ENs. In conclusion, those compounds targeting the central acidic residues and the catalytic lysine have high potential for broad-spectrum inhibitors, but if the interaction with the active site is also based on the chelation of the two metal ions in the active site (like di-ketoacid inhibitors) the possible adverse effect of transcription enhancement should be considered.

Besides the residues implicated in catalysis, here we identify residues involved in substrate binding that could also be targeted by inhibitors. Mutation of these residues in the EN domain result in a range of effect from complete loss (K148A) to no changes (T129A) in the enzymatic activity. Similarly, in the context of the L protein, the effects of these mutations range from completely benign (T129A) to a dramatic loss of any L protein activity (K148A) preventing genome transcription but also genome replication. These radical phenotypes of function loss, together with results for mutations in the N terminal structural features of TOSV EN, are evidence for the requirement for strict coordination between transcription and replication activities (i.e. cap-snatching and polymerase activities) for proper function of the L protein. Thus, drugs targeting cap-snatching ENs could eventually block both replication and transcription activities of the L protein.

### Final remarks

Recently, the enzymatic characterization of RVFV phlebovirus and CCHFV nairovirus ENs was reported, showing that the RVFV EN is active whereas CCHFV is inactive *in vitro*. The authors proposed that phenuivirus ENs are His+ and nairovirus ENs are His- (50). This work defined the first 240 amino acids of the RVFV L protein N terminus as the endonuclease domain boundaries. With the structures presented here we redefine the domain boundaries to 205 amino acids, a similar size to other cap-snatching ENs (25), and demonstrate in detail, by structural and functional analysis, the His+ nature of the phenuivirus endonucleases. Furthermore, our analysis of TOSV EN substrate specificity shows the EN preferably cleaves purine rich ssRNA longer than five nucleotides. We could not detect any sequence specificity that would explain the preferential cleavage of GC reported for TOSV by high-throughput sequencing of heterologous 5′ viral mRNAs (51), perhaps because the respective study was performed with the full-length L protein. During the revision of this manuscript the structure of the RVFV cap binding domain was reported (52), and together with the structures presented here, we now have a very complete structural view of the cap-snatching machinery of phenuiviruses. Further structural and functional studies with the full-length L protein will be necessary to understand the mechanistical interplay of both domains and the RdRp for the transcription initiation.

In summary, the structure of TOSV EN illustrates the particular folding of the family of *Phenuiviridae* cap-snatching ENs, reflecting the structural diversity of sNSV ENs with implications for their mechanisms of transcription and genome replication regulation. This folding also provides new structural mechanisms for the enlargement of L proteins during evolution. The enzyme behaves as a His+ cap-snatching EN, corroborating the current classification of His+ and His- ENs. Our structures provide the basis for structure-based design of antivirals targeting TOSV, and by defining the EN domain, we pave the way for the structure determination of cap-snatching endonucleases of other very important human pathogens such as RVFV or SFTSV, and for the development of broad spectrum inhibitors for treatment of *Bunyavirales* infections.

## DATA AVAILABILITY

Atomic coordinates and structure factors for the reported crystal structures have been deposited in the Protein Data bank under accession number 6QVV, 6QW5 and 6QW0 for the structures in the apo form, in complex with DPBA and DPBA-off respectively. The sequences used for the sequence alignments of Supplementary Figure S4 can be found at Uniprot database (https://www.uniprot.org/) under the following accession numbers: Uukuniemi (Uniprot ID: P33453), RVFV (ID: P27316), Punta toro virus (ID: A0A0F6 × 2W3), Gouleaku (ID: G0Y279), Cumuto (ID: W5VGS1), Rice stripe (ID: Q85431) and Rice grassy stunt (ID: Q9JGN8). The original alignment also includes Yichang Insect virus (ID: A0A0B5KXZ2) Arumowot virus (ID: I1SV56), Bhanja virus (ID: L7UXM1), Sandfly fever sicilian virus (ID: A0A096ZSN8) and WCH/97/HN/China/2011 (ID: I0DF35) that were removed for clarity.

## Supplementary Material

gkz838_Supplemental_Files
