# Supplementary material for: Structure and function of the Toscana virus cap-snatching endonuclease
Source: Nucleic Acids Res. 2019 Oct 4;47(20):10914–30. doi: 10.1093/nar/gkz838 (PMC6847833; doi:10.1093/nar/gkz838)
Supplement: gkz838_Supplemental_Files [file gkz838_supplemental_files.zip › SupplementaryDataV5.pdf]

	Tos211 DPBA SAD	Tos211 DPBA PDB: 6QW5	Tos211 APO PDB: 6QVV	Tos211 DPBA-off PDB: 6QW0
**Space group**	$P2_12_12$	$P2_12_12$	$P2_12_12$	$P2_12_12$
**Cell dimensions (Å)**	a=101.97<br>b=106.2<br>c=58.62<br>α=β=γ= 90	a=101.96<br>b=106.21<br>c=58.62<br>α= β= γ=90	a=101<br>b=109.64<br>c=58.16<br>α=β=γ= 90	a=100.84<br>b=107.84<br>c=58.2<br>α= β= γ=90
**Resolution range (Å)**	47.1-2.0 (2.11-2.0)	47.1-1.98 (2.09-1.98)	48.18-2.4 (2.49-2.40)	47.55 -1.5 (1.53-1.50)
**Beamline**	Soleil Prox1	Soleil Prox1	ESRF id30a	Soleil Prox1
**Wavelength (Å)**	0.97934	0. 97934	0.9677	0.978
**Completeness (%)**	100 (100)	99.0 (93.1)	98.7 (99.1)	97.9 (94.7)
**Detector**	Pilatus 6M	Pilatus 6M	Eiger X 4M	Pilatus 6M
**R-meas (%)**	16.5(101.4)	17.6 (113.9)	18.2 (121.1)	7.8 (276.2)
**I/σI**	9.65 (2.28)	10 (2.2)	7.3 (1.4)	11.6 (0.6)
**Total reflections**	482539	477675	108233	555644
**Redundancy**	11 (10.8)	10.8 (9.8)	4.2 (4.4)	5.6 (5.4)
**SigAno (3.11-2.97 Å shell)**	0.94 (1)			
**Refinement Res. range (Å)**		47.1-1.99 (2.03-1.98)	48.18-2.40 (2.50-2.40)	47.33-1.50 (1.52 – 1.50)
**No. of reflections used in refinement (free reflections)**		41468 (1811)	25520 (2676)	99758 (2989)
**R-factor**		0.205 (0.336)	0. 201(0.278)	0.2098 (0.403)
**R-free**		0.241 (0.407)	0.255 (0.319)	0.2356 (0.438)
**Total atoms in the structure [1]**		3439	3503	3459
**Protein atoms**		3072	3123	3056
**Amino acid residues**		397	401	387
**Ligand atoms**		52	58	38
**Water molecules**		315	132	365
**Average B-value (Å^2)**		19.52	36.45	25.71
**Ramachandran plot favored regions**		98.45%	95.67%	98.38%
**Ramachandran plot allowed regions**		1.55%	4.33%	1.62%
**Bond distance deviations from ideal (Å)**		0.007	0.008	0.006
**Angles deviations from ideal (°)**		0.747	0.845	0.739

Supplementary Table S1. Data refinement and collection statistics. In parenthesis are indicated the values for the outer shell.

[1]Hydrogens are not included in the number of total atoms in the structure.

	<----- A T O M 1 ----->					<----- A T O M 2 ----->					
	Atom no.	Atom name	Res name	Res no.	Chain		Atom no.	Atom name	Res name	Res no.	Chain	Distance

SO_4+2 Hydrogen bonds---

1.	1002	OG1	THR	129	A	-->	3131	O4	SO4	302	A	2.57
2.	1004	N	THR	130	A	-->	3128	O1	SO4	302	A	2.87
3.	1009	OG1	THR	130	A	-->	3128	O1	SO4	302	A	2.89

SO_4+2 Non-bonded contacts--

1.	594	CB	HIS	78	A	---	3129	O2	SO4	302	A	3.50
2.	595	CG	HIS	78	A	---	3129	O2	SO4	302	A	3.78
3.	597	CD2	HIS	78	A	---	3129	O2	SO4	302	A	3.63
4.	998	CA	THR	129	A	---	3131	O4	SO4	302	A	3.45
5.	999	C	THR	129	A	---	3128	O1	SO4	302	A	3.80
6.	1001	CB	THR	129	A	---	3131	O4	SO4	302	A	3.31
7.	1002	OG1	THR	129	A	---	3127	S	SO4	302	A	3.46
8.	1002	OG1	THR	129	A	---	3128	O1	SO4	302	A	3.36
9.	1002	OG1	THR	129	A	---	3131	O4	SO4	302	A	2.57
10.	1004	N	THR	130	A	---	3128	O1	SO4	302	A	2.87
11.	1005	CA	THR	130	A	---	3128	O1	SO4	302	A	3.50
12.	1006	C	THR	130	A	---	3128	O1	SO4	302	A	3.75
13.	1007	O	THR	130	A	---	3128	O1	SO4	302	A	3.32
14.	1008	CB	THR	130	A	---	3128	O1	SO4	302	A	3.44
15.	1009	OG1	THR	130	A	---	3128	O1	SO4	302	A	2.89
16.	1010	CG2	THR	130	A	---	3128	O1	SO4	302	A	3.50
17.	1114	CE	LYS	145	A	---	3131	O4	SO4	302	A	3.86

SO_4+3 Hydrogen bonds---

1.	1018	N	MET	132	A	-->	3133	O1	SO4	303	A	2.50

SO_4+3 Non-bonded contacts--

1.	1012	CA	THR	131	A	---	3133	O1	SO4	303	A	3.20
2.	1013	C	THR	131	A	---	3133	O1	SO4	303	A	3.28
3.	1015	CB	THR	131	A	---	3133	O1	SO4	303	A	3.90
4.	1016	OG1	THR	131	A	---	3132	S	SO4	303	A	3.81
5.	1016	OG1	THR	131	A	---	3133	O1	SO4	303	A	3.48
6.	1016	OG1	THR	131	A	---	3136	O4	SO4	303	A	3.37
7.	1018	N	MET	132	A	---	3132	S	SO4	303	A	3.74
8.	1018	N	MET	132	A	---	3133	O1	SO4	303	A	2.50
9.	1019	CA	MET	132	A	---	3133	O1	SO4	303	A	3.54
10.	1022	CB	MET	132	A	---	3133	O1	SO4	303	A	3.51
11.	1023	CG	MET	132	A	---	3133	O1	SO4	303	A	3.58

SO_4-1 Hydrogen bonds---

1.	843	N	SER	111	A	-->	3141	O4	SO4	304	A	2.84
2.	1149	OH	TYR	149	A	-->	3141	O4	SO4	304	A	2.79

SO_4-1 Non-bonded contacts--

1.	835	CA	GLN	110	A	---	3141	O4	SO4	304	A	3.49
2.	836	C	GLN	110	A	---	3141	O4	SO4	304	A	3.62
3.	838	CB	GLN	110	A	---	3141	O4	SO4	304	A	3.53
4.	843	N	SER	111	A	---	3137	S	SO4	304	A	3.64
5.	843	N	SER	111	A	---	3139	O2	SO4	304	A	3.24

6.	843	N	SER	111	A	---	3141	O4	SO4	304	A	2.84
7.	844	CA	SER	111	A	---	3141	O4	SO4	304	A	3.82
8.	845	C	SER	111	A	---	3141	O4	SO4	304	A	3.77
9.	846	O	SER	111	A	---	3141	O4	SO4	304	A	3.79
10.	847	CB	SER	111	A	---	3139	O2	SO4	304	A	3.81
11.	1136	CE	LYS	148	A	---	3138	O1	SO4	304	A	3.28
12.	1136	CE	LYS	148	A	---	3141	O4	SO4	304	A	3.85
13.	1137	NZ	LYS	148	A	---	3138	O1	SO4	304	A	3.55
14.	1137	NZ	LYS	148	A	---	3140	O3	SO4	304	A	3.76
15.	1146	CE1	TYR	149	A	---	3141	O4	SO4	304	A	3.25
16.	1148	CZ	TYR	149	A	---	3141	O4	SO4	304	A	3.43
17.	1149	OH	TYR	149	A	---	3137	S	SO4	304	A	3.72
18.	1149	OH	TYR	149	A	---	3138	O1	SO4	304	A	3.47
19.	1149	OH	TYR	149	A	---	3141	O4	SO4	304	A	2.79

SO_4′ Hydrogen bonds---

1.	1115	NZ	LYS	145	A	-->	3143	O1	SO4	305	A	2.58

SO_4′ Non-bonded contacts--

1.	1103	CB	ASN	144	A	---	3146	O4	SO4	305	A	3.89
2.	1113	CD	LYS	145	A	---	3143	O1	SO4	305	A	3.22
3.	1114	CE	LYS	145	A	---	3143	O1	SO4	305	A	3.06
4.	1115	NZ	LYS	145	A	---	3142	S	SO4	305	A	3.73
5.	1115	NZ	LYS	145	A	---	3143	O1	SO4	305	A	2.58
6.	1115	NZ	LYS	145	A	---	3144	O2	SO4	305	A	3.81
7.	1135	CD	LYS	148	A	---	3145	O3	SO4	305	A	3.90

Supplementary Table S2. List of contacts for the sulphate ions close to the active site in the apo structure of TOSV EN. Calculation performed by the PDBsum server (1). The list indicates the atom numbers and names in the PDB, the residues numbers and names and the contact distances in angstroms (Å). Hydrogen bonds and non-bonded contacts are provided separately for each sulphate ion.

Mutant	Renilla luciferase activity (sRLU) % of wild-type[2]			Signal-to-noise ratio[3]	RNA expression level (Northern blot signal) Antigenome level % of wild-type[4]	mRNA-to-antigenome ratio, relative to wild-type[5]
**D91A**	**30.99**	**+/–**	**3.70**	**932.9**	**138.4**	**0.17**
**D111A**	**3.36**	**+/–**	**<1**	**95.4**	**72.1**	**0.04**
**K143A**	**5.38**	**+/–**	**<1**	**146.9**	**136.6**	**0.04**
T127A	111.63	+/–	7.57	3347.5	101.1	1.38
T127S	145.28	+/–	51.08	3557.4	154.2	1.19
K146A	1.66	+/–	<1	54.8	6.9	0.82
K146R	220.43	+/–	7.63	6420.3	166.3	1.38
Y147A	<1	+/–	<1	1.0	1.9	0.65
Y147E	<1	+/–	<1	1.7	8.6	0.32
Y147F	132.95	+/–	32.68	3591.8	267.6	0.49
VD41/42KK	<1	+/–	<1	7.1	0.8	2.52
VD41/42SS	9.88	+/–	3.66	339.9	7.4	0.89
**ID69/70KK**	**18.81**	**+/–**	**5.24**	**511.4**	**83.2**	**0.49**
ID69/70SS	88.72	+/–	29.62	2203.5	133.1	0.88
Ndel5	<1	+/–	<1	0.6	3.2	1.88
Ndel10	<1	+/–	<1	2.1	1.7	3.43
Nscr5	15.00	+/–	4.61	439.9	18.6	1.20
Nscr10	1.22	+/–	<1	39.4	5.3	2.31
Ntos10	130.69	+/–	25.37	3369.6	113.4	1.18
IL4/5SS	<1	+/–	<1	4.7	7.2	0.29
IL4/5DD	<1	+/–	<1	1.2	5.3	0.59

Supplementary Table S3. Functional analysis of L protein mutants in the RVFV minireplicon system.

[1] For each mutant, four to five independent transfection experiments were performed. Renilla luciferase values represent mean with standard deviation (n = 4-5). Northern blots were performed once per mutant. A selective defect in mRNA synthesis was defined as reduction in Renilla luciferase level (<40%) despite wt like antigenome synthesis (>50%) and reduction of the mRNA-to-antigenome ratio (<50%). Mutants with a selective defect in mRNA synthesis are shown in boldface on grey background.

[2] Standardized relative light unit (sRLU) value (wt L protein = 100%).

[3] sRLU value of mutant divided by sRLU value of negative control mutant containing a mutation in the catalytic site of the RNA-dependent RNA polymerase.

[4] Antigenome signals in Northern blots were quantified via intensity profiles using ImageJ2 software (wt L protein = 100%).

[5] RNA signals in Northern blots were quantified using ImageJ2 software and the mRNA-to-antigenome signal ratio was calculated. The wt ratio was set at 1 for each experiment (i.e. the signal ratio of a mutant was normalized with the wt ratio) to render independent experiments comparable.

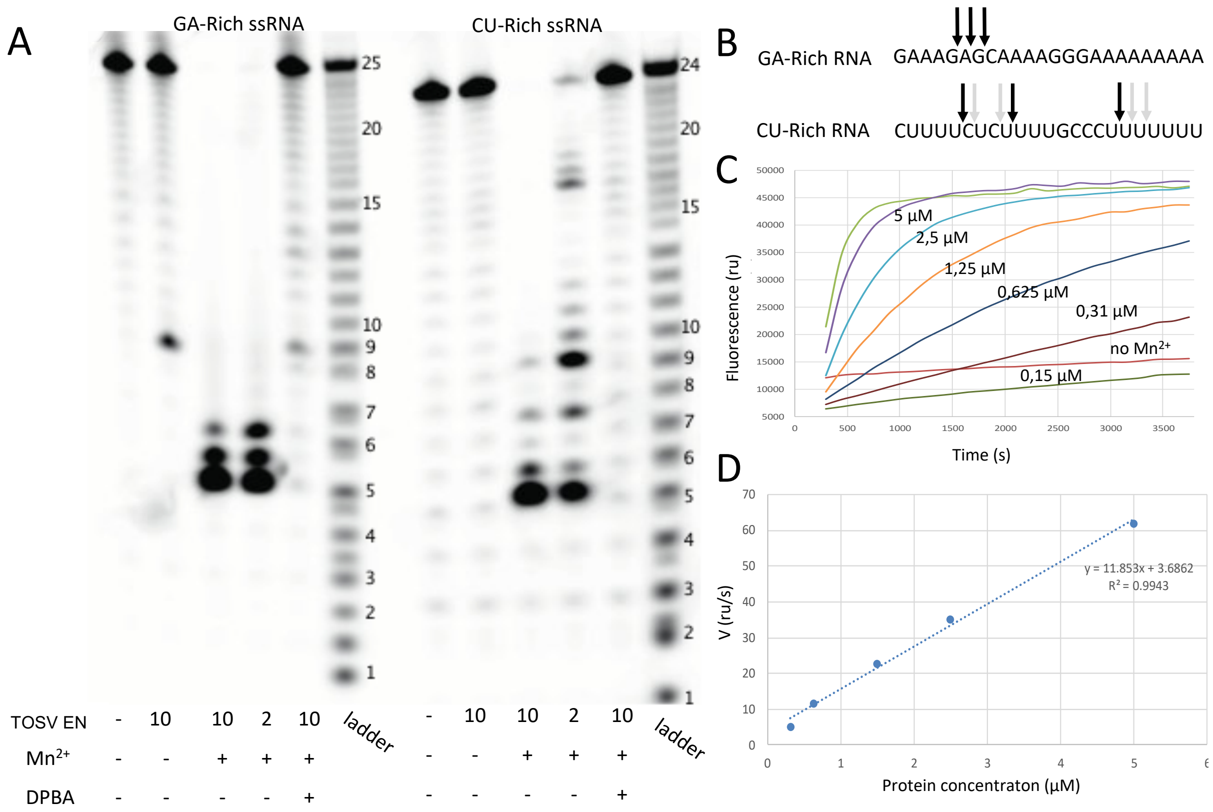

Supplementary Figure S1. Sequence specificity and kinetics of TOSV EN catalysis. **(A),** High resolution urea gel of 5' FAM fluorescently labeled GA (purine) and CU (pyrimidine) rich ssRNAs. The RNA ladder was generated by alkaline lysis of the substrates. The digestion pattern allows for identification of preferential cleavage sites. **(B)**, RNA sequences with preferential cleavage sites indicated with dark arrows (strong preference) and grey arrows (weak preference) from experiment of panel A. No sequence specificity is appreciated. **(C)**, Real time FRET experiment of the EN (as in figures 5 and 6 in reference (2) showing the raw kinetics at different enzyme concentrations. **(D)**, Plot of calculated reaction speeds vs protein concentration. The slope of the curve (11,8 ru/s•µM) determines the processivity of the enzyme.

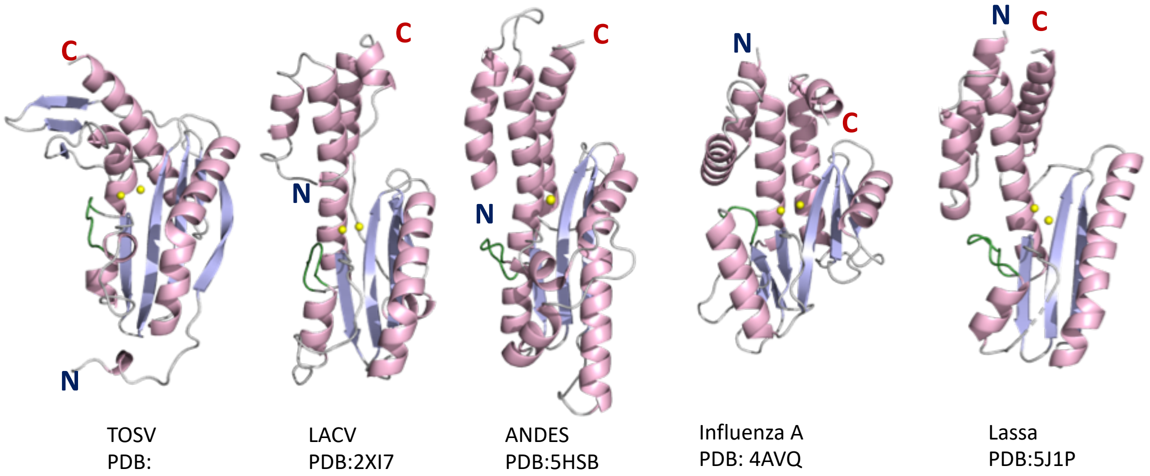

Supplementary Figure S2. Comparison of cap-snatching EN structures representative of different families of sNSVs. *Bunyavirales* TOSV (family *Phenuiviridae*), LACV (family *Peribunyaviridae*), Andes virus (family *Hantaviridae*) and Lassa virus (family *Arenaviridae*), and the Orthomyxovirus Influenza A. The PDB codes are indicated. The ENs are represented as cartoons with pink alpha helices, light blue beta sheets and grey loops. The metal ions in the active site are represented by yellow spheres. The flexible loop, present in all ENs, is highlighted in green. The loop appears in the active conformation for TOSV, LACV and Influenza, and an inactive conformation for Andes and Lassa virus. Please note that the bi-lobular fold is maintained in TOSV, despite the folding rearrangements in its N terminus.

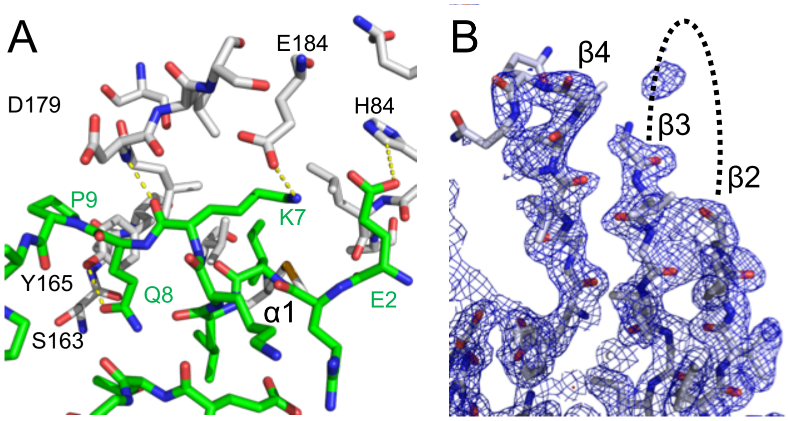

Supplementary Figure S3. (A), Crystallographic contacts of the α1 helix (green C atoms) and the symmetry related molecule (grey C atoms) of the DPBA-off structure. The nitrogen atoms are in blue and oxygen in red. The residues involved in the contacts are labelled. The hydrogen bonds at the interface are marked by dashed yellow lines. These contacts are present in all structures provided in this article. **(B)**, same representation of the TOSV N terminal beta sheet, beta strands are labelled and the 2FcFo electron density map is shown as a blue mesh (sigma 1). The β2-β3 loop lacking electron density is highlighted by a dashed black.

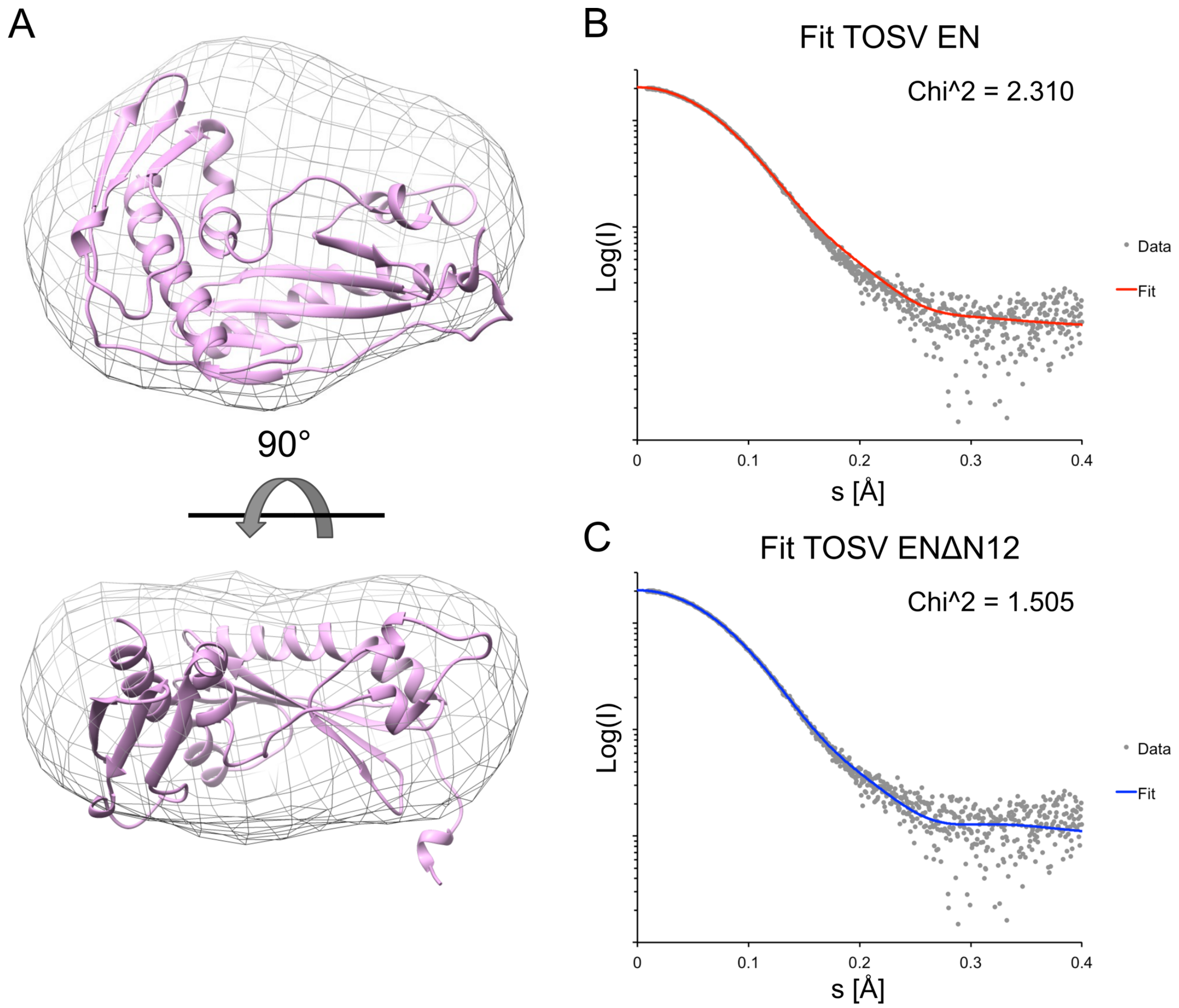

Supplementary Figure S4. Analysis of TOSV EN by Small angle X-ray scattering. TOSV EN protein was analysed by Small angle X-ray scattering (SAXS) (see supplementary methods). **(A)**, Superimposition of the SAXS-derived molecular shape with the TOSV EN monomer from the apo crystal structure (ribbon diagram) demonstrates a monomeric conformation of the protein at 9 mg/ml in solution. The figure was created using UCSF Chimera. **(B)** and **(C)**, Comparison of the experimental scattering curve of TOSV EN (grey dots) and theoretical scattering curves for the complete TOSV EN monomer from the apo crystal structure (B, red line) and the TOSV EN monomer with a deletion of the N terminal 12 residues (C, TOSV ENΔN12, blue line). χ2-values are given. The theoretical curve was calculated and fit to the experimental data using CRYSOL (3).

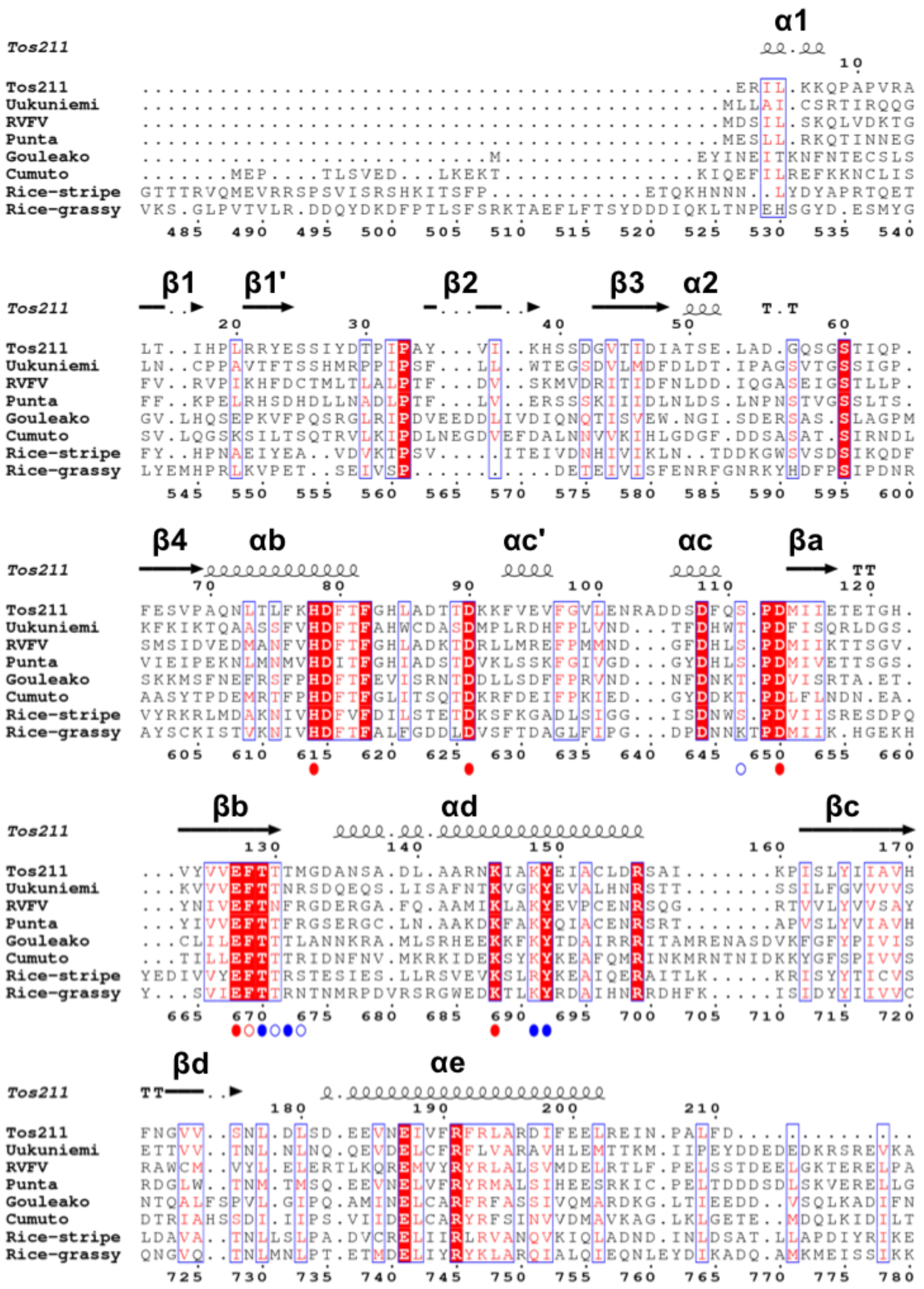

Supplementary Figure S5. Conservation of the TOSV EN domain among *Phenuiviridae* family members. The amino acid sequence alignment of the EN domains of phenuiviruses is performed with Tcoffee server (https://www.ebi.ac.uk/Tools/msa/tcoffee/) and represented by ESPript server (http://espript.ibcp.fr/ESPript/ESPript/). The alignment includes viruses from the genus Phlebovirus: TOSV, Uukuniemi, RVFV and Punta Toro virus, viruses from the genus Gokiovirus: gouleaku and cumuto virus and viruses from the genus Tenuivirus: Rice stripe and Rice grassy stunt viruses. Secondary structures of TOSV EN are indicated at the top of the sequence with the same nomenclature used in Figure 2. The amino acid numbering of TOSV and Rice grassy stunt viruses are

indicated in the top and the bottom of the alignment respectively. The catalytic residues are highlighted by red circles and sulphate ions binding residues by blue circles. Filled circles and empty circles correspond to side chain and main chain mediated contacts respectively.

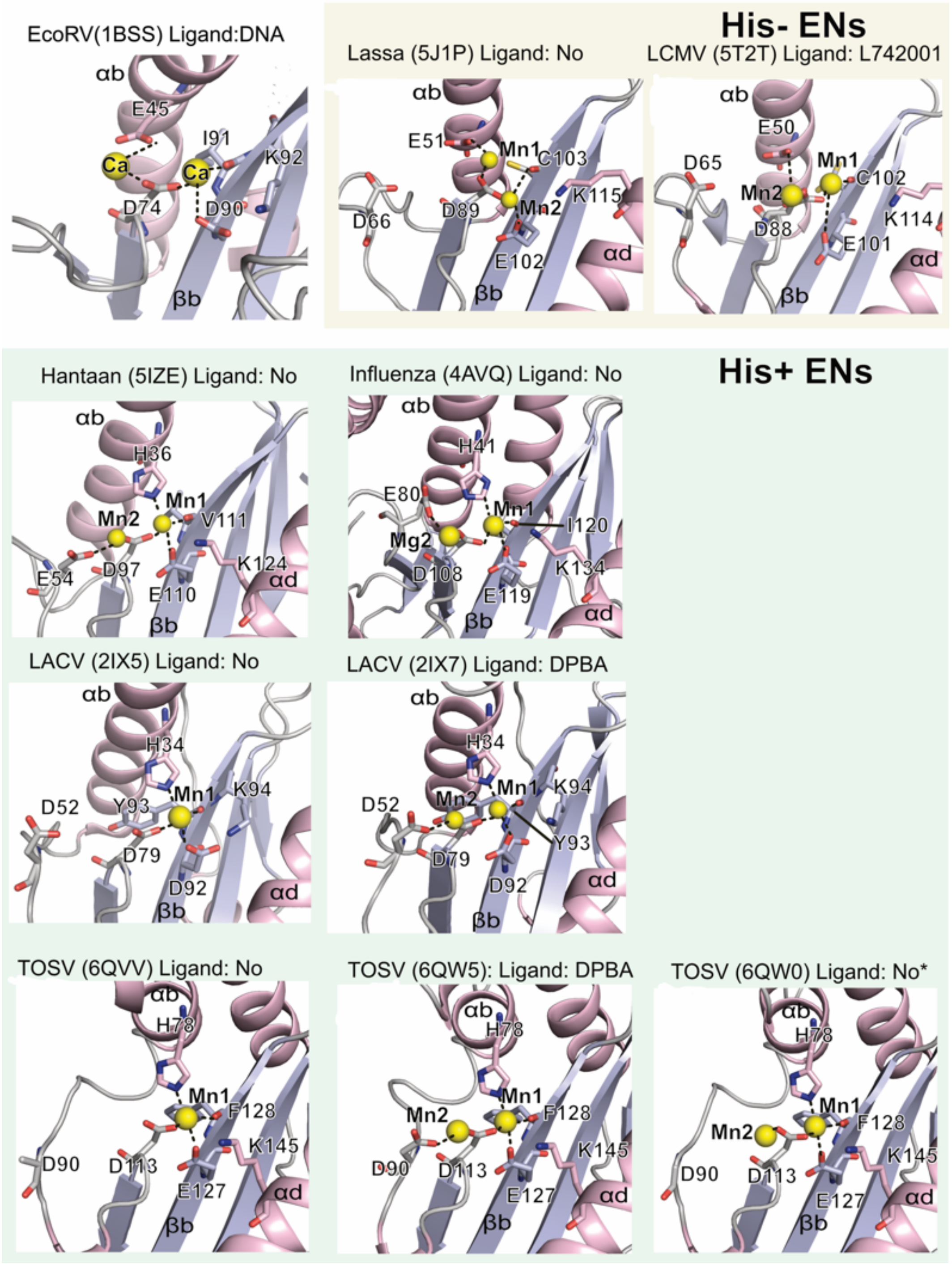

Supplementary Figure S6. Comparison of EcoRV active site structure and representative structures of His+ and His- cap-snatching endonucleases. Cartoon representation of the active sites colouring secondary structures in grey (loops) light pink (alpha helices) and light blue (beta strands). The catalytic residues are labelled and shown in sticks. The catalytic ions are shown as yellow spheres and labelled. The PDB codes are indicated for each structure. For the structures including ligands the ligands are not shown but are indicated. The active site residues of EcoRV are conserved in all cap-

snatching ENs. The His- EN active sites are similar to EcoRV. The structure of Lassa virus in complex with two metal ions shows a non-canonical metal ion binding if compared with the His+ endonucleases and with LCMV endonuclease in complex with L742001 compound. His+ cap-snatching ENs incorporate a histidine instead of the acidic residue present in His- ENs and EcoRV in helix αb. In addition, in His+ ENs an acidic residue from a flexible loop coordinates the Mn2 in the apo form (Hantaan or Infuenza) or only in the presence of a di-ketoacid inhibitor (DPBA for LACV and TOSV).

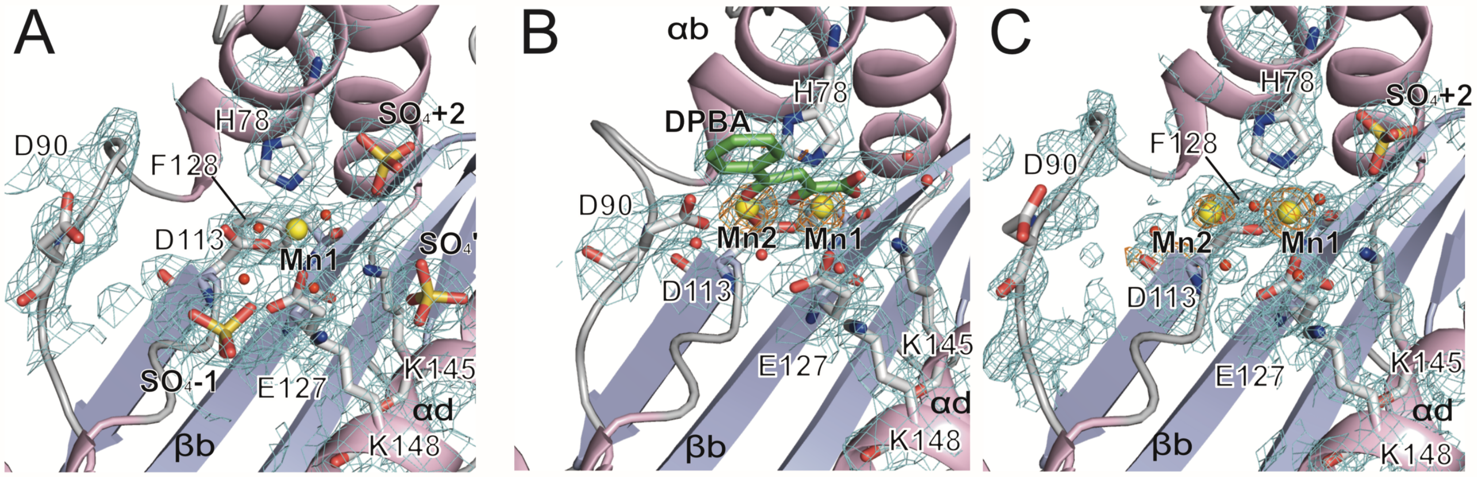

Supplementary Figure S7. Electron density of the 2FcFo map (cyan mesh) at sigma 1 and the anomalous maps as orange mesh represented at sigma 3 for the respective panels of Figure 4. **(A)**, electron density and anomalous maps for Figure 4A. **(B)**, the same for Figure 4B. **(C)**, the same for Figure 4C.

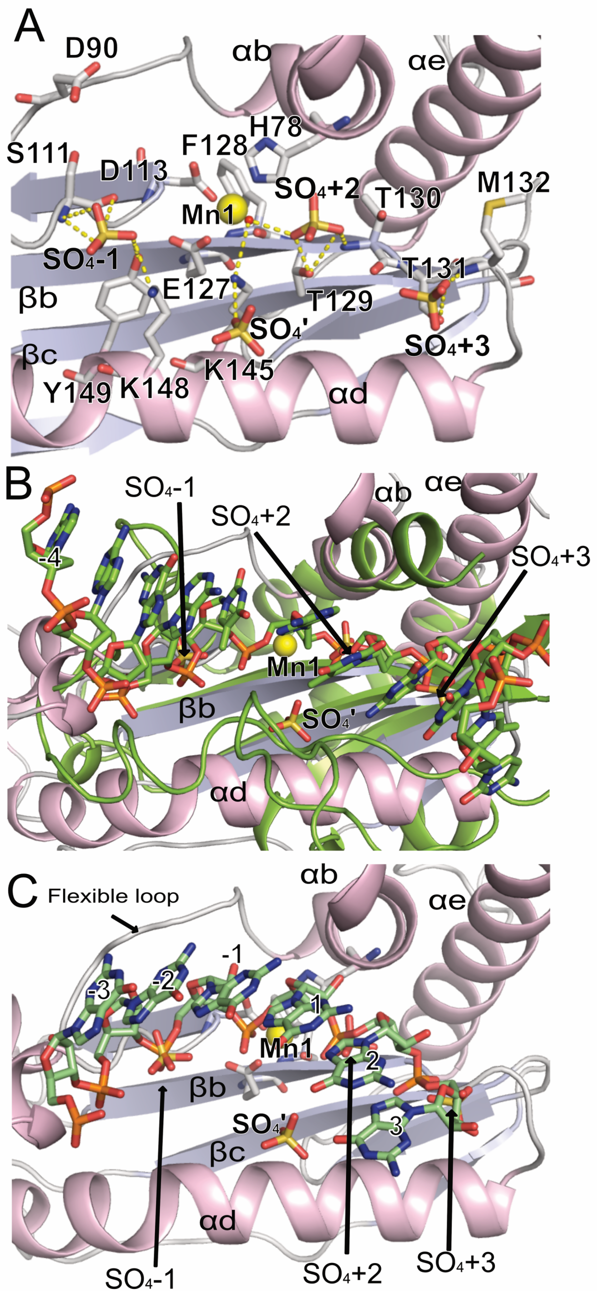

Supplementary Figure S8. Sulphate ions binding to TOSV EN mimic nucleic acids binding. **(A)**, View of the active site area including four sulphate ions. The sulphate ions binding residues are shown in sticks as well as the active site residues. Hydrogen bonds are drawn as dashed yellow lines. **(B)**, Superposition of EcoRV EN in complex with dsDNA substrate on TOSV EN showing only one strand of the dsDNA molecule in the structure. The figure shows how the sulphate ions found in the TOSV EN apo structure overlay with the DNA phosphates. The numbering of the DNA bases is shown with respect to the cleavage site at position 1. **(C)**, Superposition of the phosphates of an ssRNA molecule in the A form with the sulphate ions bound to TOSV EN. The spacing between the phosphates of the ssRNA is consistent with the spacing of the sulphate ions in the structure.

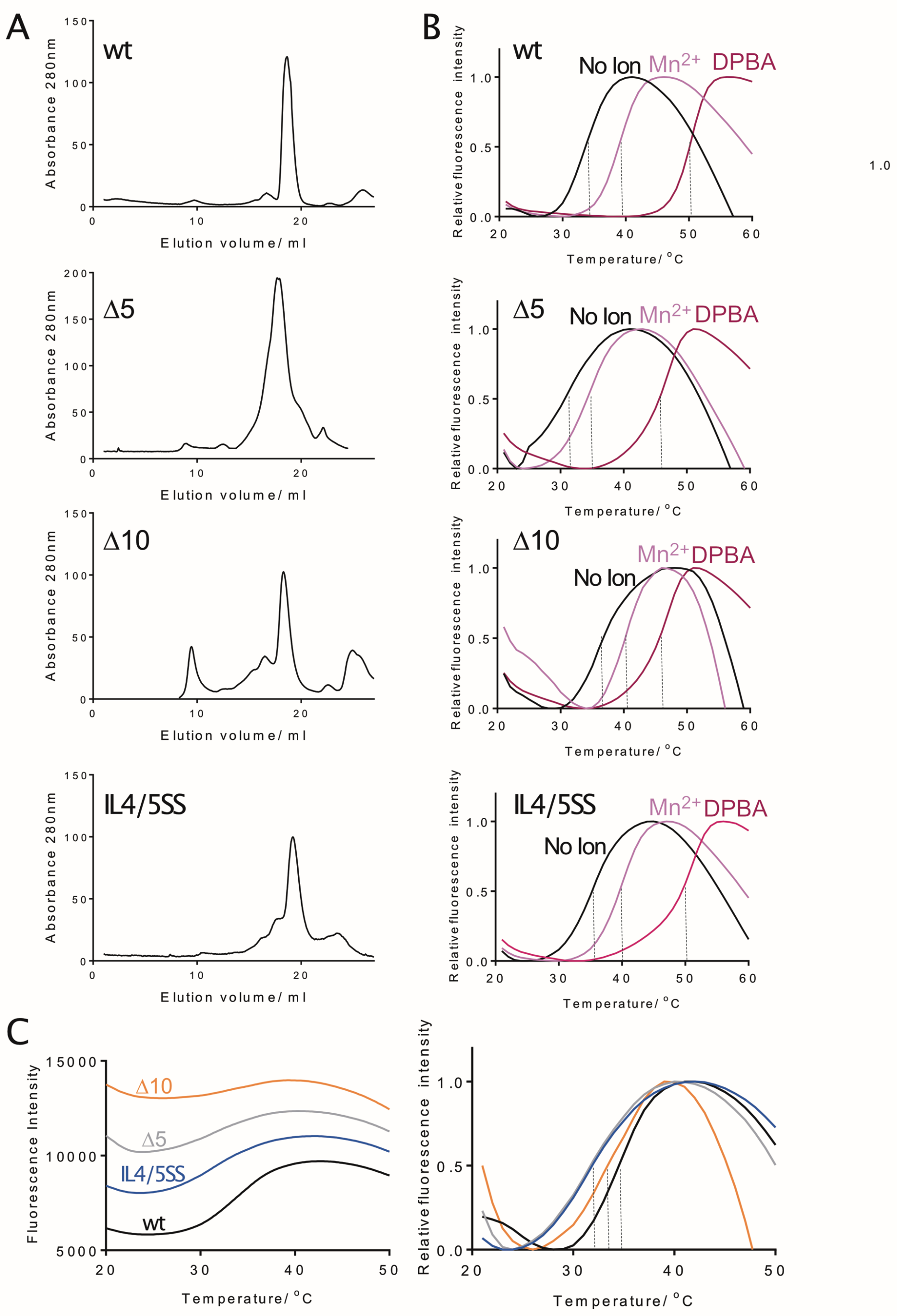

Supplementary Figure S9. Stability of N terminal truncation mutants Δ5 and Δ10, and double mutant IL4-5SS. **(A)**, Size-exclusion chromatography profiles of the mutants on a Superdex 200 10/30 column (GE Healthcare) compared to the wt protein. **(B)**, Representative normalised TSA melting curves for the wt protein and mutants without ions (black), in the presence of 2mM MnCl2 (pink) and 2mM MnCl2 and 0.5mM DPBA (burgundy). The Tm values were inferred from the inflexion points of the curves and are indicated in Table 1. **(C)**, Representative raw melting curves (right panel) and normalised melting curves (left panel) for the mutants Δ5 (grey), Δ10 (orange), IL4-5SS (blue) and wt EN (black).

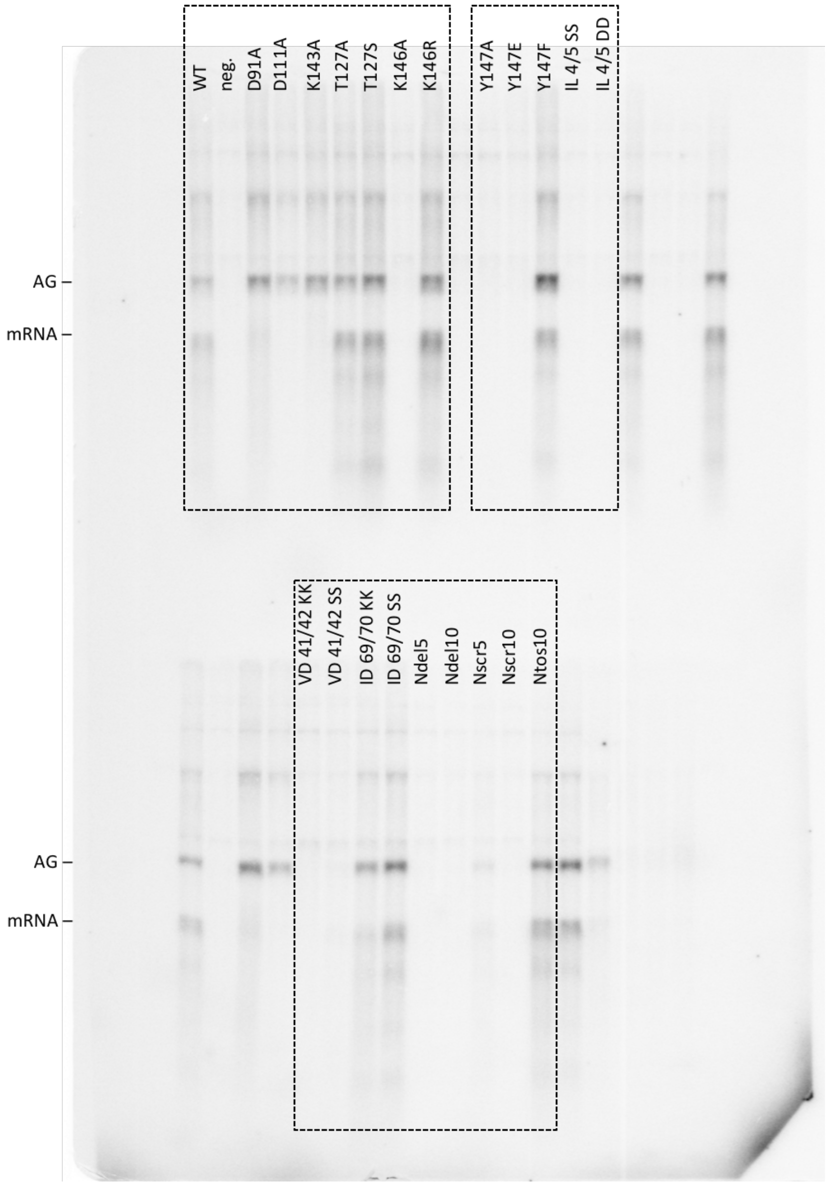

Supplementary Figure S10. Original northern blot membrane. The original autoradiogram of the northern blot presented as part of Figure 6 is shown. Lanes presented in Figure 6 are framed by a dotted rectangle and labelled with the respective L protein mutant. Positions of antigenomic RNA and mRNA bands are indicated.

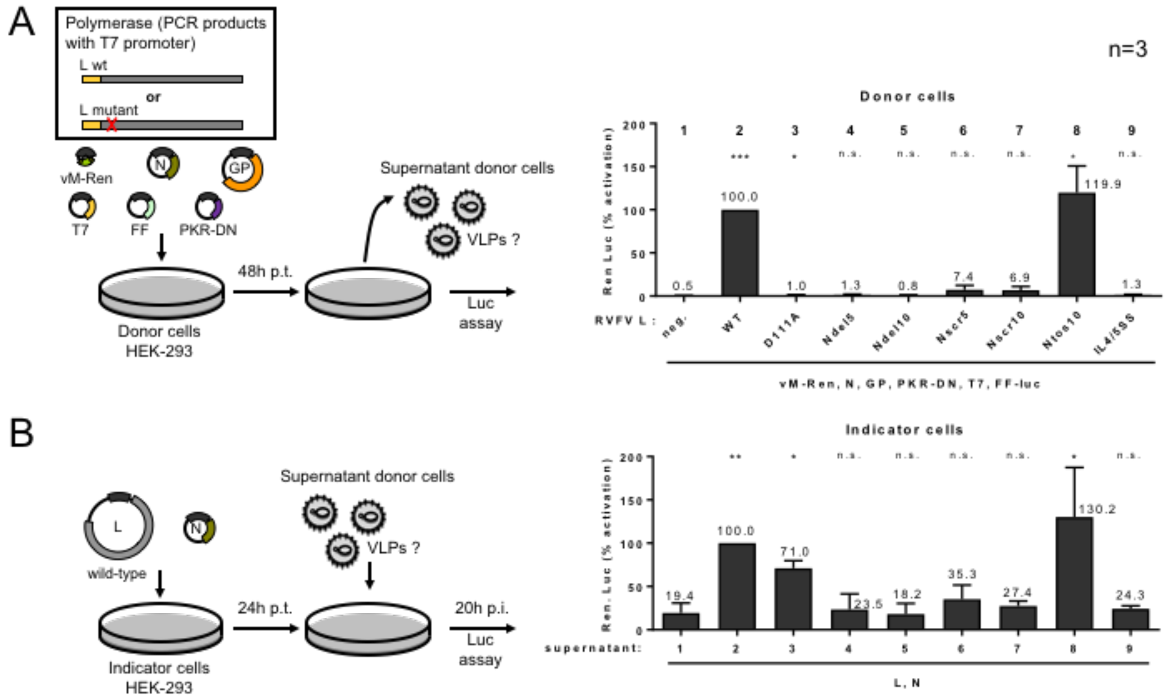

Supplementary Figure S11. Functional analysis of RVFV L protein N terminal mutations by VLP based minireplicon assay**. (A)**, Specific RVFV REN-luc minigenome activities in HEK-293 donor cells transfected with RVF VLP system. HEK-293 cells were transfected with PCR products containing a T7 promoter and the sequence of RVFV polymerase: RdRp-inactive (neg.), wt, endonuclease-inactive (D111A), or different mutants of the TOSV N terminus (Figure 6). The transfection also includes plasmids encoding the nucleoprotein N, the glycoproteins GP, a RVFV-specific REN Luc minigenome, and a T7 polymerase. A plasmid constitutively expressing Firefly luciferase (FF) served as a transfection control, and the plasmid expressing the dominant-negative form of PKR was used to boost expression. At 48h post-transfection (pt), supernatants were collected and luciferase activities were read in cell lysates. Luciferase counts were normalized on the RdRp-inactive (neg.) condition and expressed as a percentage relative to the wt L protein condition (wt).

(B), Specific RVFV REN-luc minigenome activities in HEK-293 indicator cells incubated with donor cell supernatants. The presence of VLPs in donor cell supernatants was detected by transfer of supernatants on HEK-293 indicator cells expressing wt L and N, and measurement of REN-Luc and FF-Luc activities 20 h post-infection (p.i.). The supernatants used are labeled from 1 to 9 as indicated in panel A. Normalization was done as in donor cells. Mean values and the standard deviations of 3 independent experiments are shown. A Student’s t-test was used for statistics to compare all conditions to the RdRp-inactive polymerase.

Supplementary Methods

Size-exclusion chromatography (SEC) coupled with Small-angle X-ray Scattering (SEC-SAXS). SEC-SAXS data for TOSV wt sample were collected at the SWING beamline, Synchrotron SOLEIL. In line SEC-SAXS were performed using an HPLC equipped with the BioSec3-300 column and 30 µl TOSV wt (9 mg/ml) was loaded onto at a flow rate of 300 µl/min before data collection. Scattering images with an exposure of 1 s were collected every 0.01 s using an Eiger 4M (Dectris) detector at a photon energy of 12 keV (λ = 1.033 nm) and sample to detector distance of 2 m. The experiment was performed at 10 °C. Beamline SWING-specific software was used for subtracting background buffer signal from protein signal, with a previous averaged data. Data were analysed using the ATSAS 2.8.3 package (4). The fitting to the theoretical scattering patterns from the TOSV EN crystal structure with the experimental scattering curve were calculated using CRYSOL (3). Ab initio reconstructions were generated with the program DAMMIF (5). Twenty independent DAMMIF runs were superimposed by SUPCOMB (6) and averaged using the program DAMAVER (7). The resulting model was refined using DAMMIN (8).

RVFV polymerase mutants and virus-like particle system

The required plasmids and the production of RVF virus-like particles were described previously (9,10). In order to screen several mutants, the RVFV L was expressed from PCR products containing a T7 promoter. Briefly, HEK-293 "donor" cells were reverse-transfected (TransIT-LT1, Mirus) in 6-well plates with 250ng of PCR product encoding RVFV-L (wild-type, RdRp-inactive, mutants), 500ng of pCAGGS_T7, 250ng pI.18_RVFV_N + 250ng pHH21_RVFV_vM_Ren + 250ng pI.18_RVFV_M + 125ng pI.18HA_PKR_DN + 25ng pGL3-Luc (constitutive Firefly luciferase, FF). Cell supernatants were collected 48h post-transfection, and REN-Luc and FF-Luc activities were measured in cell lysates using the dual-luciferase reporter assay system (Promega) and a Berthold TriStar2 LB942 luminometer with injector system. The supernatants were treated with 25 U/ml Benzonase (Novagen) at 37°C for 3 h and centrifuged at 12,000xg for 5 min to remove cellular debris. The presence of VLPs was validated by infecting HEK-293 "indicator" cells that were previously transfected with 250ng pI.18_RVFV-L-wt and 250ng pI.18_RVFV_N. The cells were first washed with phosphate-buffered saline (PBS) and then incubated with donor cell supernatants for 1 h at 37°C, before fresh medium containing FCS was added. Luciferase activities were measured 20 h later as described above. All renilla luciferase values were normalized on the RdRp-inactive mutant value (neg.) and expressed as a percentage of L wt value. The

two-tailed, paired Student *t* test was used to compare the different conditions to the negative control (neg.). The *P* value was considered significant if below 0.05.

Supplementary References

1. Laskowski, R.A. (2001) PDBsum: summaries and analyses of PDB structures. *Nucleic Acids Res*, **29**, 221-222.

2. Reguera, J., Gerlach, P., Rosenthal, M., Gaudon, S., Coscia, F., Gunther, S. and Cusack, S. (2016) Comparative Structural and Functional Analysis of Bunyavirus and Arenavirus Cap-Snatching Endonucleases. *PLOS Pathog*, **12**, e1005636.

3. Svergun, D., Barberato, C. and Koch, M.H.J. (1995) CRYSOL - A program to evaluate x-ray solution scattering of biological macromolecules from atomic coordinates. *Journal of Applied Crystallography*, **28**, 768-773.

4. Franke, D., Petoukhov, M.V., Konarev, P.V., Panjkovich, A., Tuukkanen, A., Mertens, H.D.T., Kikhney, A.G., Hajizadeh, N.R., Franklin, J.M., Jeffries, C.M. *et al.* (2017) ATSAS 2.8: a comprehensive data analysis suite for small-angle scattering from macromolecular solutions. *J Appl Crystallogr*, **50**, 1212-1225.

5. Franke, D. and Svergun, D.I. (2009) DAMMIF, a program for rapid ab-initio shape determination in small-angle scattering. *J Appl Crystallogr*, **42**, 342-346.

6. Kozin, M.B. and Svergun, D.I. (2001) Automated matching of high- and low-resolution structural models. *Journal of Applied Crystallography*, **34**, 33-41.

7. Volkov, V.V. and Svergun, D.I. (2003) Uniqueness of ab initio shape determination in small-angle scattering. *Journal of Applied Crystallography*, **36**, 860-864.

8. Svergun, D.I. (1999) Restoring low resolution structure of biological macromolecules from solution scattering using simulated annealing. *Biophys J*, **76**, 2879-2886.

9. Klemm, C., Reguera, J., Cusack, S., Zielecki, F., Kochs, G. and Weber, F. (2013) Systems to establish bunyavirus genome replication in the absence of transcription. *J Virol*, **87**, 8205-8212.

10. Habjan, M., Penski, N., Wagner, V., Spiegel, M., Overby, A.K., Kochs, G., Huiskonen, J.T. and Weber, F. (2009) Efficient production of Rift Valley fever virus-like particles: The antiviral protein MxA can inhibit primary transcription of bunyaviruses. *Virology*, **385**, 400-408.
